# Synthesis, Computational Studies, Antioxidant and Anti-Inflammatory Bio-Evaluation of 2,5-Disubstituted-1,3,4-Oxadiazole Derivatives

**DOI:** 10.3390/ph16071045

**Published:** 2023-07-24

**Authors:** Sibghat Mansoor Rana, Muhammad Islam, Hamid Saeed, Hummera Rafique, Muhammad Majid, Muhammad Tahir Aqeel, Fariha Imtiaz, Zaman Ashraf

**Affiliations:** 1 Punjab University College of Pharmacy, Allama Iqbal Campus, University of the Punjab, Lahore 54590, Pakistan; sibghat2971@gmail.com (S.M.R.); hamid.pharmacy@pu.edu.pk (H.S.); farihaaimtiaz@gmail.com (F.I.); 2 Department of Chemistry, University of Gujrat, Gujrat 50700, Pakistan; humera_rafique@uog.edu.pk; 3 Faculty of Pharmacy, Hamdard University Islamabad, Islamabad 45500, Pakistan; majidpharma808@gmail.com (M.M.); tahiraqeelmalik@gmail.com (M.T.A.); 4 Department of Chemistry, Allama Iqbal Open University, Islamabad 44000, Pakistan

**Keywords:** 1,3,4-oxadiazoles, synthesis, computational studies, antioxidant activity, anti-inflammatory activity

## Abstract

The 1,3,4-oxadiazole derivatives **Ox-6a-f** have been synthesized by incorporating flurbiprofen moiety with the aim to explore the potential of target molecules to decrease the oxidative stress. The title compounds **Ox-6a-f** were prepared by simple reactions in which a flurbiprofen –COOH group was esterified with methanol in an acid-catalyzed medium, which was then reacted with hydrazine to afford the corresponding hydrazide. The acid hydrazide was then cyclized into 1,3,4-oxadiazole-2-thiol by reacting with CS_2_ in the presence of KOH. The title compounds **Ox-6a-f** were synthesized by the reaction of an –SH group with various alkyl/aryl chlorides, which involves an *S*-alkylation reaction. The structures of the synthesized **Ox-6a-f** derivatives were ascertained by spectroscopic data. The in silico molecular docking was performed against target proteins cyclooxygenase-2 COX-2 (PDBID 5KIR) and cyclooxygenase-1 COX-1 (PDBID 6Y3C) to determine the binding affinity of the synthesized compounds with these structures. It has been inferred that most of the synthesized compounds bind well with an active binding site of 5KIR compared to 6Y3C, and especially compound **Ox-6f** showed excellent binding affinity (7.70 kcal/mol) among all synthesized compounds **Ox-6a-f**. The molecular dynamic (MD) simulation has also been performed to check the stability of docking complexes of ligands with COX-2 by determining their root mean square deviation and root mean square fluctuation. Little fluctuation was observed in case of **Ox-6f,** which forms the most stable complex with COX-2. The comprehensive antioxidant potential of the synthesized compounds has been evaluated by determining their free radical scavenging activity, including DPPH, OH, nitric oxide (NO), and iron chelation assay. The derivative **Ox-6f** showed promising results with 80.23% radical scavenging potential at a dose of 100 µg/mL while ascorbic acid exhibited 87.72% inhibition at the same dose. The anti-inflammatory activity of the final products has also been performed, and inflammatory markers were assayed, such as a thiobarbituric acid-reducing substance, nitric oxide, interleukin-6 (IL-6), and COX-2. The derivatives **Ox-6d** and **Ox-6f** displayed higher anti-inflammatory activity, exhibiting 70.56% and 74.16% activity, respectively. The results were compared with standard ibuprofen, which showed 84.31% activity at the same dose, 200 µg/mL. The anti-inflammatory potential has been performed by following the carrageen-induced hind paw edema model, and results showed that derivative **Ox-6f** exhibited 79.83% reduction in edema volume compared to standard ibuprofen, which reduced 84.31% edema volume. As dry lab and wet lab results confirm each other, it has been deduced that derivative **Ox-6f** may serve as the lead structure to design potent compounds to address oxidative stress.

## 1. Introduction

The reactive oxygen species, i.e., free radicals when present in cells, result in the oxidative stress which destroys lipids, DNA, proteins, and carbohydrates. These active oxygen species are either endogenous and/or exogenous and destroy the biomolecules, ultimately changing the organism’s structure and functions [1,2]. The destruction of tissues is because of various enzymes and some other non-enzymatic reactions, which actually produced highly reactive species. This trauma, which is produced as a result of oxidative stress, is known to be intricate in lifestyle-changing disorders, including multiple sclerosis cardiovascular, diabetes mellitus, ischemic diseases, malignancies, Alzheimer’s disease, or Parkinson’s disease [3,4,5].

Azoles are five-member heterocycles which contain at least one nitrogen atom along with the presence of other atoms. This class of nitrogen heterocycles has enormous applications in the field of medicinal chemistry; substituted azole derivatives have been reported to possess anti-inflammatory activity, pain-killing effects, carbonic anhydrase inhibitory activity, and blood pressure-lowering properties [6,7,8]. A number of substituted oxadiazole analogues have muscle relaxant action, anxiolytics, and tumor cell growth inhibitors [9]. The presence of heteroatoms in azoles assists this moiety to form the strong drug receptor interactions which ultimately increase its biological activities. The acetylcholine esterase and alkaline phosphatase inhibitory potential of triazole and thiadiazole derivatives have been reported in literature [10]. An important member of the azole family is 1,3,4-oxadiazole, which is part of many compounds having pharmacological potential, such as GSK-3β inhibitors with in vivo antidepressant activity [11], antiviral [12,13], antimicrobial [14], enzyme inhibitory activity [15], anti-proliferative and antioxidant activities [16,17], anti-malarial, anti-cancer and antimicrobial activities [18,19], and β-glucuronidase inhibitory activity [20]. A number of azole derivatives act as muscle relaxants and stop the cell division in cancer cells. The clinically used drugs which possess this heterocyclic moiety are raltegravir, nesapidil, and the antibiotic Furamizole. 

Some of the important characteristics of 1,3,4-oxadiazole derivatives are their water solubility, lower lipophilicity, and better metabolic stability compared to other isomeric oxadiazoles. This nucleus can also be used as a bioisostere with the replacement of different functionalities like esters, amides, and carbamates. The aromatic nature of oxadiazole and its planar geometry help this moiety to act as a flat aromatic linker to provide appropriate orientation to bind at COX-2 enzymes, which decrease inflammation without producing ulcerogenic effects [21]. A number of synthetic methodologies to construct substituted 1,3,4-oxadiazoles have been reported in the recent literature, which assured the versatile applications of this heterocyclic ring system [22,23,24,25]. The transition metal-catalyzed formations of different functionalities have been prepared, which are important precursors for the preparation of heterocycles [26,27,28,29,30].

Keeping in view the enormous pharmacological applications of the 1,3,4-oxadiazole nucleus, the current research work was designed to synthesize the 1,3,4-oxadiazole derivatives by incorporating the flurbiprofen moiety. Computational molecular docking studies were performed to determine the binding interactions of the designed molecules with target protein. The MD simulation has been performed to determine the stability of the protein-ligand complex by calculating their root mean square deviation and root mean square fluctuations. The antioxidant activity of synthesized 1,3,4-oxadiazole derivatives **Ox-6a-f** was carried out to ascertain the potential of these derivatives to decrease oxidative stress. The in vivo anti-inflammatory activity of the prepared oxazole derivatives **Ox-6a-f** was also determined, and inflammatory markers such as nitric oxide (NO), thiobarbituric acid-reducing substance (TBARS), interleukin-6 (IL-6), and cyclooxydenae-2 (COX-2) have been well studied, and results were compared with standard ibuprofen. 

## 2. Results and Discussion

### 2.1. Synthesis

**Scheme 1:**
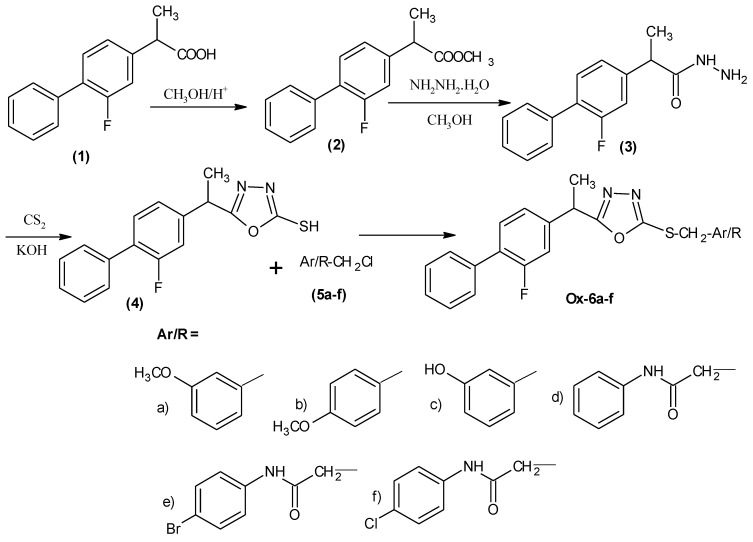
Oxadiazole derivatives **Ox-6a-f**.

The methyl ester of flurbiprofen **2** was successfully prepared by reacting a carboxylic group of flurbiprofen with anhydrous methanol in the presence of a catalytic amount of concentrated H_2_SO_4_. The ester formation is confirmed by disappearance of hydroxyl stretching absorption in the region around 3400 cm^−1^ in the FTIR spectrum. The flurbiprofen ester was then reacted with hydrazine, which furnished the hydrazide **3** in excellent yield. The cyclization of the hydrazide **3** was accomplished by reacting it with CS_2_ and KOH to afford the intermediate 1,3,4-oxadiazole **4**. The formation of each product was ascertained by the FTIR spectral data, and the stretching absorption appeared in the acceptable region in corresponding functional groups. The FTIR spectrum of hydrazide has a stretching absorption of –NH and –NH_2_ at 3345 cm^−1^ and 3366 cm^−1^, respectively. The formation of the intermediate oxadiazole **4** was ascertained by the presence of the peak at 1587 cm^−1^, which reflects the C=N absorption and SH at 2853 cm^−1^. The title compounds **Ox-6a-f** were synthesized by reacting oxadiazole **4** and substituted alkyl chlorides **5a**-**f**. This involves a nucleophilic attack of the SH group on alkyl halide, i.e., alkylation of the mercapto group of oxadiazole (**4**). Scheme 1 shows the synthetic route for the preparation of the final products **Ox-6a-f**. The structures of the synthesized oxadiazole derivatives **Ox-6a-f** have been confirmed by spectral data, i.e., FTIR, ^1^H NMR, and ^13^C NMR spectroscopy. The NMR spectra of the synthesized compounds have been given in supplementary materials.

### 2.2. Molecular Docking Studies

**Figure 1:**
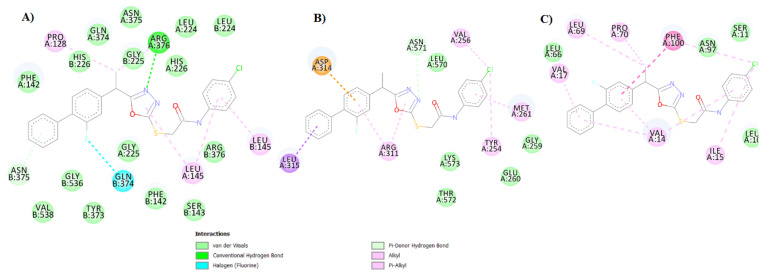
Docking interactions of the oxadiazole derivative **Ox-6f** with target proteins (**A**) cyclooxygenase-2 (PDBID 5KIR), (**B**) cyclooxygenase-1 (PDBID 6Y3C), and (**C**) NADPH reductase (PDBID 7U8G).

The docked complexes of prepared 1,3,4-oxadiazole derivatives **Ox-6a-f** were analyzed based on the lowest binding energy values (kcal/mol) and hydrogen/hydrophobic bonding analyses. The reliability and efficacy of target proteins COX-2 (PDB ID 5KIR), COX-1 (PDB ID 6Y3C), and NADPH oxidase NOX-2 (PDBID 7U8G) were confirmed by Ramachandran plots given in supporting information (Figures S1–S3). The Ramachandran plots showed that 96.5% of amino acid residues were in favored (97%) regions and 100.0% residues were in allowed regions. These Ramachandran graph values exhibited good accuracy of phi (φ) and psi (ψ) angles among coordinates of proteins, and most of the residues plummeted in acceptable region. It has been observed that oxadiazole derivatives exhibited more potential to inhibit the COX-2 as compared to the COX-1 enzyme. The results showed that **Ox-6f,** among the synthesized oxadiazole derivatives, was the most active compound with the best binding energy value (−7.70 kcal/mol) compared to other derivatives in Table 1. The docking pose of the most potent derivative **Ox-6f** was selected on the basis of energy value and the interaction patterns of ligands within the active region of target proteins (Figure 1). It has been found that heterocyclic oxadiazole rings are involved in binding interactions with ARG376 and pi stacking interactions with LEU 145. The anilide phenyl ring also forms pi stacking with LEU145, and flurbiprofen fluorine interacts with the amino acid GLN374 of target protein COX-2. The conformational position within the active region of the target protein was confirmed by superimposition.

### 2.3. Molecular Dynamic Simulation Studies

**Figure 2:**
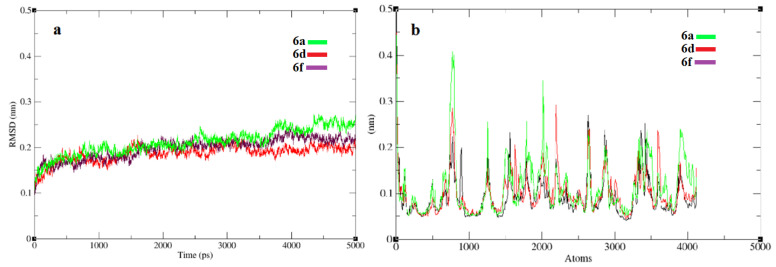
Molecular dynamic simulation of protein–ligand complexes, (**a**) the root mean square deviation plot, and (**b**) the root mean square fluctuation plot of the protein–ligand complexes. The green represents the complex for **Ox-6a,** red for **Ox-6d,** and purple for **Ox-6f** against COX-2.

In comparison with the structural flexibility behavior of the COX-2 protein, three compounds with the highest binding affinity were incorporated in Gromacs 4.5.4 and MD simulation studies were performed for 10 ns with these three complexes. The root mean square deviation of COX-2 complexes was determined against their original structure in the ligand-protein complex, and plots were generated using Xmgrace software. There is little fluctuation in the overall period of simulation of all three complexes. The MD simulation also assures that the binding of **Ox-6f** in the active binding site of COX-2 protein forms a stable complex and also does not disturb the backbone of the target protein (Figure 2a). The root mean square fluctuation has also been calculated to find out the residual mobility of the protein–ligand complex. The graphs were plotted against residual atomic numbers based on the trajectory period of the simulation. It has been confirmed that small residual fluctuation was observed in the cases of **Ox-6a** and **Ox-6d,** while in the case of **Ox-6f,** the fluctuation was further decreased (Figure 2b). 

### 2.4. In Vitro Antioxidant Assays

**Figure 3:**
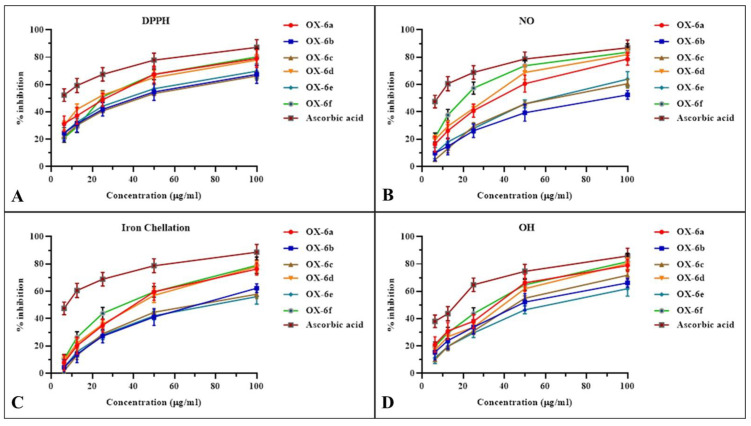
In vitro antioxidant activities of oxadiazol derivatives **Ox-6a-f** (**A**) DPPH radical scavenging activity, (**B**) NO● scavenging activity, (**C**) OH● scavenging activity, and (**D**) iron chelating % inhibition. Each value represents the mean ± SD (n = 3).

To assess the antioxidant potential of oxadiazole derivatives, multimode antioxidant assays were performed. In DPPH assay, **Ox-6f** exhibited excellent antioxidant potential, displaying 80.23% inhibition of the free radical at 100 µg/mL concentration. The antioxidant activity results were compared with ascorbic acid (87.21%), which was used as a positive control in this bioassay. The IC_50_ value of the most potent derivative, **Ox-6f,** was found to be 25.35 µg/mL, while for ascorbic acid the IC_50_ value was 6.13 µg/mL. The presence of 4-chlorophenyl moiety of anilide in derivative **Ox-6f** plays a very important role in antioxidant activity. The nitric oxide free radical scavenging potential also assured that **Ox-6f** is the most active derivative among all other derivatives, showing 83.88% inhibition of the NO free radical. The **Ox-6d** also exhibited good antioxidant activity with 81.96% scavenging ability of NO free radicals compared to standard ascorbic acid (86.71%). The IC_50_ calculated for the most active compound **Ox-6f** was 27.32 µg/mL, while for ascorbic acid, the IC_50_ was 19.81 µg/mL. The most potent derivative **Ox-6f** possesses multiple sites like a biphenyl ring system, hetero aryl oxadiazole moiety, and para disubstituted anilide residue, which help to stabilize the free radicals, thus showing good antioxidant activity. The free radical, when produced at the aromatic ring system, can be stabilized by delocalization of free radicals in a different position. The **Ox-6d** also has different aromatic units, which can accommodate the free radicals and increase their stability due to the presence of conjugation. These structural features in compounds **Ox-6f** and **Ox-6d** are important in showing good antioxidant activities. The hydroxyl free radical inhibition results showed that compound **Ox-6f** and **Ox-6d** have the highest activity with 81.69% and 80.28% inhibitions of OH radicals at 100 µg/mL concentration compared to the standard gallic acid (85.91%) at the same concentration. The antioxidant activity of synthesized oxadiazole derivatives **Ox-6a-f** was also determined in terms of iron chelating power of these compounds. The results of iron chelation ability also support our previous finding showing that **Ox-6f** possesses the highest activity and chelates 78.94% of iron in FeCl_3_, having IC_50_ 32.82 µg/mL. The comprehensive antioxidant activity results are summarized in Figure 3.

### 2.5. In Vitro Anti-Inflammatory Potential

**Figure 4:**
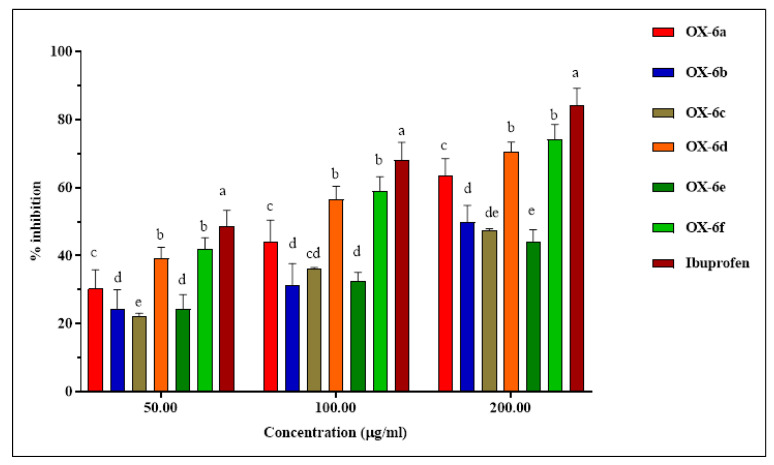
In vitro anti-inflammatory assessment of oxadiazole derivatives **Ox-6a-f** via albumin denaturation. Each value is represented as mean ± SD (n = 3). Means with different superscript (a–e) letters in the column are significantly (*p* < 0.05) different from one another.

The in vitro anti-inflammatory activity of the synthesized oxadiazole derivatives **Ox-6a-f** was determined with the help of heat-induced albumin denaturation assay. The oxadiazole derivative **Ox-6f** displayed the most promising anti-inflammatory activity with percent inhibition (74.16 ± 4.41%) compared to standard drug ibuprofen (84.31 ± 4.93%) at a concentration of 200 μg/mL. The presence of oxadiazole moiety along with the presence of side chain *p*-chlorophenyl substitution is vital in showing the anti-inflammatory potential of derivative **Ox-6f**. Besides the most potent derivative, **Ox-6f**, synthesized oxadiazole derivatives **Ox-6d** and **Ox-6a** also displayed good anti-inflammatory activity, having 70.56 ± 2.87% and 63.66 ± 4.91% inhibitory potential, respectively. The presence of the 3-methoxy-substituted phenyl ring in compound **Ox-6a,** along with the 2,5-disubstitution pattern of 1,3,4-oxadiazole moiety, plays a very important role in displaying good anti-inflammatory activity. The compound **Ox-6d** has structural similarities with most the potent derivative, **Ox-6f,** as both possess the anilide moiety which is vital in showing anti-inflammatory activity. The most potent derivative **Ox-6f** has a chloro-substituted phenyl ring, while the compound **Ox-6d** has an unsubstituted phenyl ring, and the rest of the functional groups are common in both compounds. The in vitro anti-inflammatory activity results of the synthesized oxadiazole derivatives **Ox-6a-f** are presented in Figure 4. 

### 2.6. In Vivo Anti-Inflammatory Potential

The in vivo anti-inflammatory activity of target molecules has also been evaluated by following the carrageenan-induced rat paw edema model. The reduction in rat paw edema in the presence of synthesized oxadiazoles **Ox-6a-f** is shown in Table 2. Based upon in vivo anti-inflammatory results, it has been concluded that **Ox-6f** possessed the highest potential to decrease edema volume with significance (*p* < 0.05). The most potent compound **Ox-6f** at a dose of 10 mg/kg of the body weight of rats significantly reduced the carrageenan-induced edema up to 79.830 ± 4.04%. The reduction in edema volume by the standard drug ibuprofen at the same dose was 84.71 ± 2.77% after 12 h of carrageenan injection. The oxadiazole derivatives **Ox-6d,** bearing anilide moiety, and **Ox-6a,** possessing methoxy phenyl substitution, also showed good anti-inflammatory activity with 76.64 ± 3.21% and 74.52 ± 3.97% inhibition of edema after 12 h, respectively.

### 2.7. Effect on Antioxidant Enzymes

Table 3 enlists the expressed concentrations of antioxidant enzymes in hind paw homogenates of rats treated with synthesized compounds **Ox-6a-f**. The endogenous antioxidant enzyme levels of only **Ox-6e**-containing derivatives showed some alterations, indicating a mild range of toxicity in vivo. On the other hand, the paramount reservation (*p* < 0.05) of antioxidant enzymes in all tested groups is indicative of their safer nature. Significant reservation of endogenous antioxidants suggests that oxadiazole derivatives, specially **Ox-6f** and **Ox-6d,** reduce carrageenan-induced oxidative stress and counter down infiltration of prostaglandins and chemokines.

### 2.8. Effect on Inflammatory Markers

**Figure 5:**
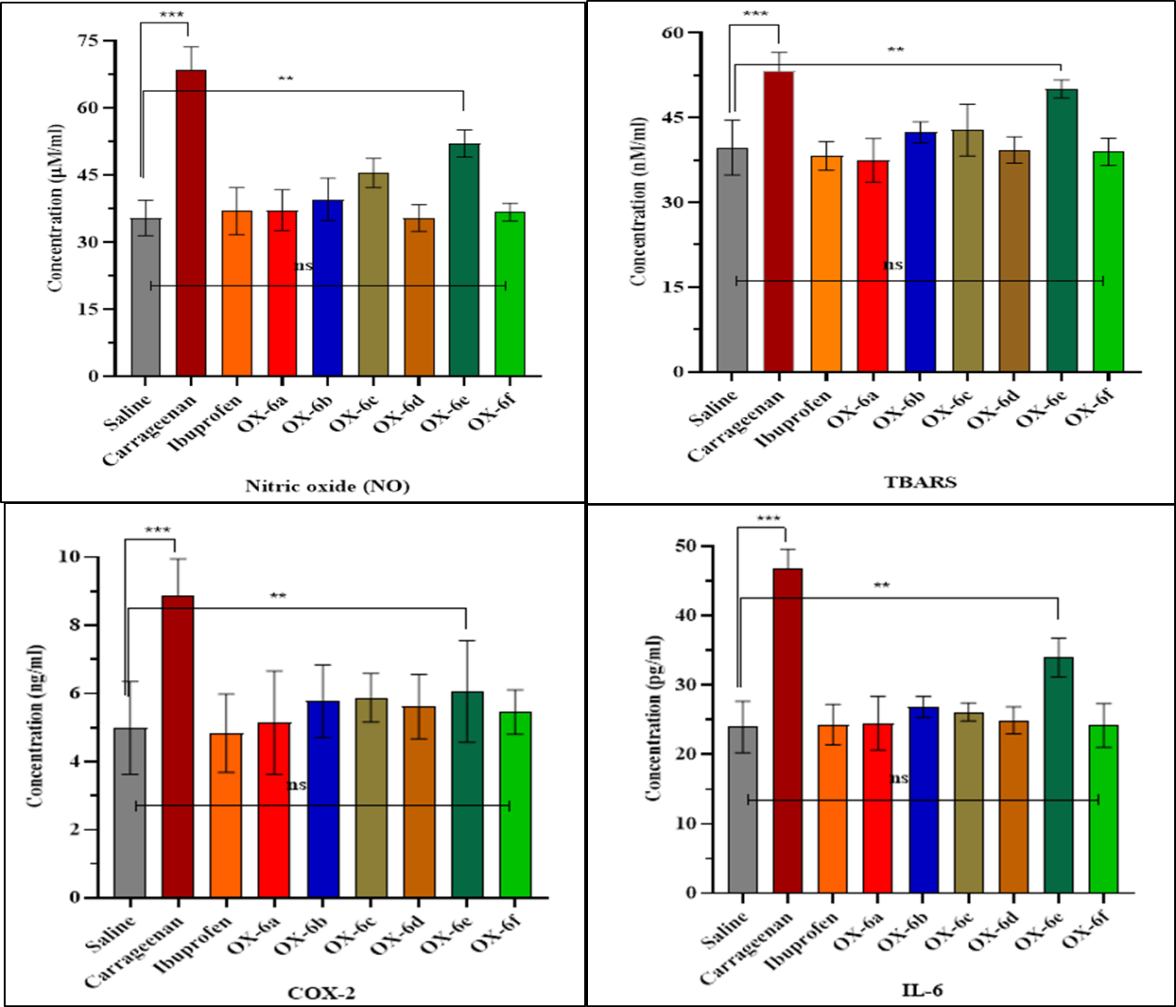
Pro-inflammatory markers in experimental animals after the administration of oxadiazole derivatives Ox-6a-f (10 mg/kg). (NO) Nitric oxide, (TBARS) Thiobarbituric acid-reducing substances (Lipid peroxidation assay), (IL-6) Interleukin-6, (COX-2) Cyclooxygenase 2. Data is presented as mean ± SD (n = 3). Differences in serum levels were considered significant at the level of ** *p* < 0.05 and *** *p* < 0.01.

The substantial reduction in paw edema of test groups treated with synthesized oxadiazoles **Ox-6a-f** is attributed to their substantial anti-inflammatory potential that can be observed in the serum levels of pro-inflammatory markers (Figure 5). Obvious suppression of carrageenan-induced NO, TBARS, IL-6, and COX-2 concentrations can be observed in the paw homogenates of test groups, indicating the anti-inflammatory nature of the **Ox-6a-f** derivatives. The results showed that derivative **Ox-6f** possesses the highest activity of inflammatory biomarkers, reflecting its greater anti-inflammatory potential. Based upon our results, it may be concluded that derivative **Ox-6f** acts as potential candidate to develop clinical drugs to address the oxidative stress.

### 2.9. Materials and Methods

All chemicals used for the synthesis of compounds were purchased from Sigma-Aldrich Chemical Co., St. Louis, MO, USA. Melting points were determined using a Digimelt MPA 160, USA melting point apparatus. ^1^H NMR and ^13^C NMR spectra were determined as CDCl_3_ solutions at 300 MHz and 100 MHz, respectively, on a Bruker AM-300 machine, Mundelein, IL 60060 USA. FTIR spectra were recorded using Shimadzu FTIR-8400S spectrometer (Kyoto, Japan, cm^−1^), and elemental analyses were performed with a LECO-183 CHNS analyzer, Vouersweg 118, Geleen, The Netherlands.

#### 2.9.1. Synthesis of Intermediate 1,3,4-Oxadiazole (**4**)

The 2-(2-fluorobiphenyl-4-yl)propanoic acid (**1**) (2.054 mmol) was mixed with 30 mL of anhydrous methanol in a round bottom flask, and 2–4 drops of the concentrated sulfuric acid were also added into the reaction flask. The reaction mixture was refluxed for 6 h; progress of the reaction was monitored by TLC. After the completion of the reaction, the excess of methanol was removed under reduced pressure and added to 100 mL of water. The methyl 2-(2-fluorobiphenyl-4-yl)propanoate (**2**) was extracted with ethyl acetate. The formation of ester was ascertained by the disappearance of –OH absorption and presence of C=O stretching absorption at 1725 cm^−1^. The ester (**2**) (1.937 mmol) was dissolved into 30 mL of ethanol and hydrazine (3.875 mmol) was added into the reaction mixture. The mixture was refluxed for 4 h, and upon the completion of the reaction monitored by TLC, the ethanol was evaporated using a rotary evaporator. The residue was then poured into 50 mL of ice-cold water to precipitate 2-(2-fluorobiphenyl-4-yl)propanehydrazide (**3**). The presence of hydrazide moiety in the structure of hydrazide was confirmed by the presence of –NH and -NH_2_ absorption at 3345 cm^−1^ and 3366 cm^−1^, respectively, in the FTIR spectrum. The intermediate 5-[1-(2-fluorobiphenyl-4-yl)ethyl]-1,3,4-oxadiazole-2-thiol **(4)** was synthesized by reacting hydrazide (**3**) (10 mmol) with CS_2_ (20 mmol) in the presence of KOH (5 mmol) and dry ethanol (30 mL) in a round bottom flask [31]. The CS_2_ was added into the reaction mixture in portions and was refluxed for 12 h. Completion of the reaction was assured by TLC, and after the completion of the reaction, the mixture was concentrated under reduced pressure and was then acidified with HCl to afford oxadiazole (**4**) as yellow precipitates. The crude intermediate (**4**) was purified by recrystallization in ethanol to afford crystals; yield: 74%; **FTIR** (KBr, ν_max_ cm^−1^): 2932 (sp^2^ C-H stretch), 2867 (sp^3^ C-H stretch), 1622 (C=N), 1587 (C=C aromatic), 1223 (C-O).

#### 2.9.2. Synthesis of Substituted 1,3,4-Oxadiazole Derivatives Ox-6a-f

The title compounds **Ox-6a-f** were prepared by reacting intermediate 1,3,4-oxadiazole (**4**) with alkyl chlorides, 1-(chloromethyl)-3-methoxybenzene (**5a**), 1-(chloromethyl)-4-methoxybenzene (**5b**), 3-(chloromethyl)phenol (**5c**), 2-chloro-*N*-phenylacetamide (**5d**), *N*-(4-bromophenyl)-2-chloroacetamide (**5e**), and *N*-(4-chlorophenyl)-2-chloroacetamide (**5f**) by following the already-reported method with slight modification [32]. Briefly 1,3,4-oxadiazole (**4**) (3.5 mmol) was mixed with KOH (3.5 mmol) solution in 15 mL of water with continuous stirring for about 5 min. The alkyl chlorides (**5a**–**f**) (3.5 mmol) were then dissolved into acetone (10 mL) and added slowly into the reaction mixture. The reaction mixture was then stirred up to the completion of the reaction as monitored by TLC. The excess of the solvent was evaporated using a rotary evaporator, and the reaction mixture was treated with HCl to change the pH to neutral. The precipitates formed in the reaction flask were filtered and washed with water and purified by recrystallizing with 95% ethanol to give pure final products **Ox-6a-f**.

#### 2.9.3. Synthesis and Characterization of 2-(3-Methoxybenzylsulfanyl)-5-[1-(2-fluorobiphenyl-4-yl)ethyl]-1,3,4-oxadiazole Ox-6a

Physical data: Yield: 72%; solid m.p. 182–184 °C, R: 0.28 (pet. ether: ethyl acetate 2:1), **FTIR** (KBr-ν_max,_ cm^−1^): 2966 (sp^2^C–H stretch), 2873 (sp^3^C–H stretch), 1582 (C=N, stretching of thiadiazole), 1535 (C=C, Ar-stretching), 1063 (C-F). **^1^HNMR** (CDCl_3_, 300 MHz, δ ppm) data: 1.65 (3H, d, J = 6.5 Hz, CH_3_), 4.29 (s, 3H, -OCH_3_), 4.31 (1H, q, J = 6.5 Hz, H-13), 4.62 (2H, s, SCH_2_), 7.11 (1H, d, = 7.1 Hz, H-6′), 7.16 (1H, s, H-2′), 7.19 (5H, m, Phenyl ring), 7.33 (2H, m, H-4′,5′), 7.41 (2H, d, J = 7.8 Hz, H-2,6), 7.48 (1H, d, J = 7.5 Hz, H-5); **^13^C NMR** (75 MHz, CDCl_3_, δ ppm) data: 18.6 (CH_3_), 36.1 (SCH_2_), 45.7 (C–13), 61.1 (OCH_3_), 115.3 (C–2′), 115.7 (C–6′), 123.6 (C-4′), 127.0 (C-5′), 128.8 (C–8, 10, 12), 129.4 (C–9,11), 130.7 (C–6), 131.0 (C–5), 133.7 (C–1), 134.3 (C–7), 135.3 (C-4), 141.2 (^1^*J*_CF_ = 165 Hz, C–3), 158.06 (C–15,16), 159.8 (C–1′), 161.3 (C–3′); Anal Calcd For C_24_H_21_O_2_N_2_SF: C, 68.57; H, 5.00; Found C, 68.51; H, 4.92.

#### 2.9.4. Synthesis and Characterization of 2-(4-Methoxybenzylsulfanyl)-5-[1-(2-fluorobiphenyl-4-yl)ethyl]-1,3,4-oxadiazole Ox-6b

Physical data: Yield: 76%; solid m.p. 201–203 °C, R: 0.31 (pet. Ether: ethyl acetate 2:1), **FTIR** (KBr-ν_max,_ cm^−1^): 2954 (sp^2^C–H stretch), 2888 (sp^3^C–H stretch), 1604 (C=N stretching of thiadiazole), 1588 (C=C Ar-stretching), 1077 (C-F). **^1^HNMR** (CDCl_3_, 300 MHz, δ ppm) data: 1.65 (3H, d, J = 7.3 Hz, CH_3_), 4.28 (s, 3H, -OCH_3_), 4.32 (1H, q, J = 7.3 Hz, H-13), 4.50 (2H, s, SCH_2_), 7.24 (2H, d, = 7.6 Hz, H-2′,6′), 7.29 (2H, d, = 7.6 Hz, H-3′,5′), 7.31 (5H, m, Phenyl ring), 7.38 (2H, d, J = 7.2 Hz, H-2,6), 7.42 (1H, d, J = 7.2 Hz, H-5); **^13^C NMR** (75 MHz, CDCl_3_, δ ppm) data: 18.7 (CH_3_), 38.4 (SCH_2_), 45.7 (C–13), 61.1 (OCH_3_), 115.4 (C–2′,6′), 123.6 (C–3′,5′), 128.1 (C–6), 130.9 (C–8, 12), 131.0 (C–9, 10, 11), 135.4 (C–7), 135.8 (C-1), 141.2 (C–1′), 141.3 (C-2), 158.0 (C-4), 160.0 (C-5), 161.0 (^1^*J*_CF_ = 185 Hz, C–3), 161.3 (C-15, 16), 172.3 (C-4′); Anal Calcd For C_24_H_21_O_2_N_2_SF: C, 68.57; H, 5.00; Found C, 68.49; H, 4.94.

#### 2.9.5. Synthesis and Characterization of 2-(3-Hydroxybenzylsulfanyl)-5-[1-(2-fluorobiphenyl-4-yl)ethyl]-1,3,4-oxadiazole Ox-6c

Physical data: Yield: 67%; solid m.p. 192–193 °C, R: 0.25 (pet. Ether: ethyl acetate 2:1), **FTIR** (KBr-ν_max,_ cm^−1^): 3465 (-OH), 2976 (sp^2^C–H stretch), 2898 (sp^3^C–H stretch), 1591 (C=N, stretch of thiadiazole), 1539 (C=C Ar-stretching), 1058 (C-F). **^1^HNMR** (CDCl_3_, 300 MHz, δ ppm) data: 1.63 (3H, d, J = 7.1 Hz, CH_3_), 4.31 (1H, q, J = 7.1 Hz, H-13), 4.62 (2H, s, SCH_2_), 7.11 (1H, d, = 6.6 Hz, H-6′), 7.14 (s, 1H, H-2′), 7.21 (5H, m, Phenyl ring), 7.34 (2H, m, H-4′,5′), 7.41 (2H, d, J = 6.5 Hz, H-2,6), 7.482 (1H, d, J = 7.3 Hz, H-5); **^13^C NMR** (75 MHz, CDCl_3_, δ ppm) data: 18.6 (CH_3_), 36.1 (SCH_2_), 45.7 (C–13), 115.3 (C–2′), 123.6 (C–6′), 127.0 (C-4′), 128.4 (C-5′), 129.8 (C–8, 12), 130.7 (C–9,10,11), 131.0 (C–6), 133.7 (C–5), 135.3 (C–1), 141.2 (C–7), 158.0 (C-4), 159.8 (^1^*J*_CF_ = 214 Hz, C–3), 161.0 (C–15,16), 161.3 (C–1′), 172.3 (C–3′); Anal Calcd For C_23_H_19_O_2_N_2_SF: C, 67.98; H, 4.67; Found C, 67.90; H, 4.61.

#### 2.9.6. Synthesis and Characterization of 2-({5-[1-(2-Fluorobiphenyl-4-yl)ethyl]-1,3,4-oxadiazol-2-yl}sulfanyl)-N-phenylacetamide Ox-6d

Physical data: Yield: 78%; solid m.p. 214–216 °C, R: 0.24 (n-hexane:ethyl acetate 2:1), **FTIR** (KBr-ν_max,_ cm^−1^): 3321 (NH), 2989 (sp^2^C–H stretch), 2887 (sp^3^C–H stretch), 1654 (amide C=O), 1581 (C=N, stretch of thiadiazole), 1540 (C=C, Ar-stretch), 1071 (C-F). **^1^HNMR** (CDCl_3_, 300 MHz, δ ppm) data: 1.58 (3H, d, J = 6.8 Hz, CH_3_), 4.26 (1H, q, J = 6.8 Hz, H-13), 4.47 (2H, s, SCH_2_), 7.03 (2H, d, = 7.8 Hz, H-2′, 6′), 7.12 (3H, m, H-3′,4′,5′), 7.18 (5H, m, Phenyl ring), 7.24 (2H, d, J = 6.9 Hz, H-2,6), 7.25 (1H, d, J = 6.9 Hz, H-5), 7.26 (1H, s, NH); **^13^C NMR** (75 MHz CDCl_3_, δ ppm) data: 19.2 (CH_3_), 36.8 (SCH_2_), 45.8 (C–13), 115.5 (C–2′,6′), 123.6 (C–3′,4′,5′), 126.3 (C–8, 12), 128.4 (C–9,10,11), 129.9 (C–6), 130.8 (C–5), 133.2 (C–1), 135.3 (C–7), 137.0 (C-4), 141.2 (^1^*J*_CF_ = 185 Hz, C–3), 158.7 (C–15,16), 160.9 (C–1′), 172.3 (C=O); Anal Calcd For C_24_H_20_O_2_N_3_SF: C, 66.51; H, 4.61; Found C, 66.43; H, 4.54.

#### 2.9.7. Synthesis and Characterization of 2-({5-[1-(2-Fluorobiphenyl-4-yl)ethyl]-1,3,4-oxadiazol-2-yl}sulfanyl)-N-(4-bromophenylacetamide Ox-6e

Physical data: Yield: 72%; solid m.p. 228–230 °C, R: 0.26 (pet. Ether: ethyl acetate 2:1), **FTIR** (KBr- ν_max,_ cm^−1^): 3432 (NH), 2977 (sp^2^C–H stretch), 2865 (sp^3^C–H stretch), 1643 (amide C=O), 1576 (C=N, stretch of thiadiazole), 1556 (C=C, Ar-stretch), 1067 (C-F). **^1^HNMR** (CDCl_3_, 300 MHz, δ ppm) data: 1.63 (3H, d, J = 5.1 Hz, CH_3_), 4.24 (1H, q, J = 5.1 Hz, H-13), 4.45 (2H, s, SCH_2_), 7.22 (2H, d, = 6.7 Hz, H-2′, 6′), 7.24 (2H, d, = 6.7 Hz, H-3′, **5′**), 7.30 (5H, m, Phenyl ring), 7.35 (2H, d, J = 7.3 Hz, H-2,6), 7.41 (1H, d, J = 7.1 Hz, H-5), 7.47 (1H, s, NH); **^13^C NMR** (75 MHz, CDCl_3_, δ ppm) data: 18.7 (CH_3_), 36.3 (SCH_2_), 45.7 (C–13), 115.4 (C–2′,6′), 115.7 (C–3′,5′), 123.5 (C–8, 12), 127.6 (C–9,10,11), 128.2 (C–6), 128.9 (C–5), 130.9 (C–1), 135.4 (C–7), 141.3 (C-4), 158.0 (C–4′), 160.7 (^1^*J*_CF_ = 225 Hz, C-3), 160.9 (C–15,16), 161.3 (C–1′), 172.4 (C=O); Anal Calcd For C_24_H_19_O_2_N_3_SFBr: C, 56.26; H, 3.71; Found C, 56.17; H, 3.66.

#### 2.9.8. Synthesis and Characterization of 2-({5-[1-(2-Fluorobiphenyl-4-yl)ethyl]-1,3,4-oxadiazol-2-yl}sulfanyl)-N-(4-chlorophenylacetamide Ox-6f

Physical data: Yield: 71%; solid m.p. 236–238 °C, R: 0.24 (n-hexane:ethyl acetate 2:1), **FTIR** (KBr- ν_max,_ cm^−1^): 3366 (NH), 2988 (sp^2^C–H stretch), 2871 (sp^3^C–H stretch), 1661 (amide C=O), 1582 (C=N stretching of thiadiazole), 1567 (C=C Ar-stretching), 1087 (C-F). **^1^HNMR** (CDCl_3_, 300 MHz, δ ppm) data: 2.38 (3H, d, J = 6.9 Hz, CH_3_), 4.16 (1H, q, J = 6.6 Hz, H-13), 4.59 (2H, s, SCH_2_), 7.12 (2H, d, = 7.6 Hz, H-2′, 6′), 7.31 (2H, d, = 7.6 Hz, H-3′, **5′**), 7.37 (5H, m, Phenyl ring), 7.42 (2H, d, J = 6.8 Hz, H-2,6), 7.43 (1H, d, J = 6.8 Hz, H-5), 7.45 (1H, s, NH); **^13^C NMR** (75 MHz, CDCl_3_, δ ppm) data: 22.8 (CH_3_), 36.3 (SCH_2_), 45.7 (C-13), 112.6 (C–2′,6′), 115.4 (C–3′,5′), 115.7 (C–8, 12), 123.5 (C–9,10,11), 127.6 (C–6), 128.2 (C–5), 128.9 (C–1), 130.9 (C–7), 135.4 (C-4), 141.4 (C–4′), 158.0 (^1^*J*_CF_ = 170 Hz, C-3), 160.7 (C–15,16), 161.3 (C–1′), 172.4 (C=O); Anal Calcd For C_24_H_19_O_2_N_3_SFCl: C, 61.57; H, 4.06; Found C, 61.48; H, 3.97.

#### 2.9.9. In Silico Docking Studies

##### Retrieval of Proteins

The crystal structures of target proteins COX-2 (PDB ID 5KIR), COX-1 (PDB ID 6Y3C), and NADPH oxidase NOX-2 (PDBID 7U8G) were obtained from the Protein Data Bank (PDB) (http://www.rcsb.org accessed on 1 June 2022). After retrieving the protein structures, the energy minimization was done by applying the gradient algorithm and Amber force field in UCSF Chimera 1.10.1 [33]. The stereochemical properties, Ramachandran graph, and values [34] of target proteins were assessed by the Molprobity server [35], while the hydrophobicity graph was generated by Discovery Studio 4.1 Client [36]. The protein architecture and statistical percentage values of helices, beta-sheets, coils, and turns were accessed by VADAR 1.8 [37].

##### Ligand Structures and In Silico Docking

The synthesized oxadiazole derivative **Ox-6a-f** structures were sketched in the ACD/ChemSketch tool. The oxadiazoles structures were further energy minimized in stable conformations by UCSF Chimera 1.10.1. The molecular docking of oxadiazoles (Ox-1-6) was performed against COX enzymes using the diverse PyRx tool [38]. In docking experiments, the oxadiazole derivatives **Ox-6a-f** preferred to orient within the active binding sites of target proteins. To run the docking experiment, grid box parametric dimension values were adjusted as X = 61.0781, Y = 56.3001, and Z = 63.1015, whereas the centered values fixed as X = −2.9272, Y = 22.0164, and Z = −32.4883, respectively. The default exhaustiveness = 8 value was used to obtain the finest binding conformational pose of protein-ligand docked complexes. The oxadiazoles **Ox-6a-f** were docked separately against the crystal structure of target enzymes, and the obtained docked complexes were further evaluated on the lowest binding energy (kcal/mol) value and binding interactions pattern. The docked complexes were further evaluated on the lowest binding energy (kcal/mol) values, and hydrogen and hydrophobic bond analysis using Discovery Studio (4.1) and UCSF Chimera 1.10.1 [39] was accomplished.

##### Molecular Dynamics Simulations

The MD simulation studies were performed, and among all synthesized derivatives, **Ox-6a, Ox-6b,** and **Ox-6f** were selected on the basis of binding energy values. The selected best-ranked energy docked complexes **Ox-6a, Ox-6b,** and **Ox-6f** were employed for Molecular Dynamics Simulations using Gromacs 4.5.4 separately [40] in order to assure their stability against COX-2 (PDBID 5KIR). The receptor molecules were separated from docked complexes and built their topology files using GROMOS 53A6 force-field and water model SPC216. Similarly, selected molecules’ **Ox-6a, Ox-6b,** and **Ox-6f** topology files were generated using an online automated topology builder, PRODRG Server [41]. The protein–ligand complexes were then solvated with SPC216 explicit water molecules and placed in the center of a cubic box of size 24 × 24 × 24 A3. We adjusted 1.0 Å distance between COX-2 and the edge of the simulation box to immerse the protein with water and rotate freely. Prior to minimization, the overall system charge was neutralized by adding ions, and the steepest descent approach (1000 ps) was used for each protein–ligand complex for energy minimization (nsteps = 50,000). The Particle Mesh Ewald (PME) method was employed for energy calculation [42]. Further NVT was performed for 100 ps to equilibrate the system with protein and ligands for constant volume, pressure (1 atm) and temperature (300 K). The final MD run was set to 5000 ps for each protein–ligand complex with nsteps 2,500,000, and trajectories were saved for further analysis using Xmgrace and UCSF Chimera 1.10.1 software.

#### 2.9.10. DPPH Radical Scavenging Assay

The DPPH radical scavenging activity of synthesized oxadiazoles **Ox-6a-f** was determined by following the already reported method [43]. Briefly, 24 mg of free radical (DPPH) was mixed with 100 mL CH_3_OH, and this stock solution was kept at 20 °C for further utilization. The DPPH solution was diluted with methanol to optimize its optical density at 0.86 (±0.02) at 517 nm. Then, oxadiazole solutions 10 µL with different concentrations (6.25–100 μg/mL) were mixed with 190 µL of DPPH solution. The solutions were stirred and then incubated for a further 15 min at 25 °C. The optical density of the mixed solution was noted at a wavelength of 517 nm. The standard used in this study was ascorbic acid for comparison purposes. The DPPH scavenging activity of the synthesized compounds was determined as:

$$\%\;\mathrm{inhibition}=\left(\frac{abs\;control-abs\;sample}{abs\;control}\right)\times 100$$

#### 2.9.11. OH Scavenging Assay

The antioxidant potential of oxadiazoles **Ox-6a-f** was also determined by using the scavenging of hydroxyl free radicals assay. Briefly, 500 μL of 2-deoxyribose having a concentration of 2.8 mM was made in phosphate buffer (50 mM) and adjusted its pH at 7.4. To assess the antioxidant activity of synthesized oxadiazoles **Ox-6a-f,** the reaction mixture was finally prepared as 100 μL of 0.1 M EDTA, 200μL of ferric chloride 100 mM, 100 μL of 200 mM of H_2_O_2_, and 100 μL of synthesized compounds **Ox-6a-f**. The reaction was started by adding 100 μL of 300 mM of ascorbic acid, and the mixture was incubated at 37 °C for 1 h. After that, 1 mL of trichloroacetic acid 2.8% and thiobarbituric acid (1 mL) 1% in 50 mM sodium hydroxide were added to the reaction mixture. The reaction mixture was heated at 70–80 °C for 20 min, and then the temperature was decreased to 25 °C to record the absorbance at 532 nm. The OH free radical trapping potential of synthesized compounds was determined as:

$$\%\;\mathrm{inhibition}=\left(\frac{abs\;control-abs\;sample}{abs\;control}\right)\times 100$$

#### 2.9.12. Nitric Oxide Scavenging Assay

This antioxidant assay was performed to find out the NO scavenging activity of synthesized oxadiazole derivatives **Ox-6a-f**. First of all, 0.1% of napthylenediamine in distilled H_2_O was mixed with 1% of sulfanilamide in 5% phosphoric acid to prepare the Griess reagent. Then, 100 μL of oxadiazole derivatives **Ox-6a-f** were added to an equal volume of sodium nitroprusside (10 mM), which was prepared in phosphate buffer saline. A total of 1 mL of the Griess reagent was added to the reaction mixture and incubated at 25 °C for a period of 3 h, and optical density was recorded at a wavelength of 546 nm. Ascorbic acid was used as positive control in this study. The % inhibition of each sample solution was determined as:

$$\%\;\mathrm{inhibition}=\left(\frac{abs\;control-abs\;sample}{abs\;control}\right)\times 100$$

#### 2.9.13. Iron Chelation

The antioxidant activity was also determined by iron (II) binding ability, as the iron chelating ability of any compound infers its antioxidant activity. The oxadiazoles **Ox-6a-f** solution 200 μL in CH_3_OH was added into 900 μL of methanol and 100μL of iron chloride solution (FeCl_2_.2H_2_O, 2.0 mM), and the reaction mixture prepared was incubated at room temperature for 5 min. To assess the antioxidant activity, the reaction was initiated by adding 400 μL of ferrozine at a concentration of 5.0 mM. The mixture was then incubated at 25 °C for 10 min. The optical density was measured at 562 nm with the help of EDTA, which was used as a positive control to compare the results. The iron chelating capacity was determined as:

$$\mathrm{Chelation}\;\mathrm{effect}\%=\left(\frac{abs\;control-abs\;sample}{abs\;control}\right)\times 100$$

#### 2.9.14. Lipid Peroxidation (TBARS) Estimation

The lipid peroxidation activity was determined by following the already reported method with some modifications [44]. Briefly, 20 μL of ferric chloride (100 mM) and 200 μL of ascorbic acid (100 mM) were mixed to prepare the reaction mixture. The phosphate buffer was added to adjust the pH to 7.4 and make the volume up to 1000 μL. The reaction mixture was then incubated at 37 °C for 30 min. After that, 1000 μL of 10% trichloroacetic acid was added into the reaction mixture to quench the reaction. Then, the mixture was treated with 1000 μL of 0.66% thiobarbituric acid, and the reaction mixture was kept in boiling water for 20 min and the temperature gradually decreased to 25 °C. The amount of the thiobarbituric acid-reducing substance (TBARS), which reflects lipid peroxidation, was measured by recording the absorbance at wavelength 535 nm. 

#### 2.9.15. Nitrite Assay

The nitrite assay was performed to measure the amount of nitrite in the tissue samples before and after treatment with oxadiazole derivatives **Ox-6a-f**. The deproteinization of tissues was carried out by adding (100 μL) of 0.3 M NaOH and 5% ZnSO_4_ into the samples. The mixture was then centrifuged at 6000 rpm for 20 min, and 30 μL of the supernatant was taken and mixed with 2 mL of the Griess reagent. The activity was determined by recording the absorbance at wavelength 540 nm. The amount of nitrite in the tissues was determined by a sodium nitrite curve.

#### 2.9.16. Catalase Assay (CAT)

Catalase activity of the synthesized compounds **Ox-6a-f** was measured by the method reported earlier [45]. First of all, phosphate buffers with pH of 5.0 were prepared; 400 μL of H_2_O_2_ (5.9 mM) was added into the buffer solution to prepare the reaction mixture. The activity was measured by measuring the change in absorbance, which was determined at wavelength 240 nm. The absorbance was measured after each time scale, and one unit of catalase activity was demarcated as an absorbance variation of 0.01 units per minute. 

#### 2.9.17. Peroxidase Assay (POD)

Peroxidase inhibition activity of the synthesized compounds **Ox-6a-f** was measured to check the inhibitory effects on the peroxidase enzyme. The peroxidase assay was performed by preparation of the reaction mixture. The mixture contained phosphate buffer (50 mM, 2500 μL) whose pH was maintained at pH 5.0, guaiacol (20 mM) 150 μL, and 300 μL of H_2_O_2_ (50 mM). The peroxidase activity was determined by measuring the change in absorbance after each interval of time at wavelength 470 nm and one unit peroxidase activity equal to the change in absorbance of 0.01 units per minute.

#### 2.9.18. Superoxide Dismutase Assay (SOD)

The superoxide dismutase assay of the oxadiazole derivatives **Ox-6a-f** was carried out to determine the superoxide dismutase inhibitory activity of tested derivatives. The phenazine methosulphate (186 μM, 100 μL) was mixed with sodium pyrophosphate buffer 1200 μL maintained at pH 7.0 (0.052 mM). To this reaction mixture, add 300 μL of the supernatant, which was obtained from paw homogenates and centrifuged subsequently at 2000 rpm and 10,000 rpm for 10 min and 15 min, respectively. Then, 0.2 mL of NADH (780 μM) was added into the reaction mixture and reaction was quenched by adding CH_3_COOH (1000 μL). The activity was determined by measuring the absorbance at wavelength 560 nm, and results were expressed in units/mg protein.

#### 2.9.19. Reduced Glutathione Assay (GSH)

Reduced glutathione assay of the synthesized oxadiazole derivatives **Ox-6a-f** was performed. The reaction mixture was prepared by mixing 1000 μL of the supernatant of the homogenate with 4% sulfosalicylic acid 1000 μL. The reaction mixture was then centrifuged at 2000 rpm for 30 min. The 100 μL of filtrate was added into phosphate buffer (0.1 M, 2700 μL) to adjust the pH at 7.4. To this reaction mixture, 200 μL of 100 mM 1,2-dithio-bis nitro-benzoic acid (DTNB) was added, and activity was determined by measuring absorbance immediately at wavelength 412 nm. The reduced glutathione activity was determined as μM GSH/g in tissue.

#### 2.9.20. Total Protein Estimation

The total protein estimation was performed to assess the effects of oxadiazoles **Ox-6a-f** on bovine serum albumin. The paw tissues, which were homogenized, were prepared to carry out the assay by adding an appropriate amount of EDTA (1 mM solution) and phosphate buffer (100 mM solution) to maintain the pH at 7.4. The reaction mixture was kept at 4 °C for about 20 min and then centrifuged at 12,000 rpm, and the supernatant was collected. The total protein estimation was measured by following the already reported method [46] with the help of the bovine serum albumin (BSA) standard curve.

#### 2.9.21. Proinflammatory Cytokines

Tissue homogenates were prepared according to the laboratory’s standard protocol, and the total protein concentration was determined [47]. The homogenates were diluted in phosphate-buffer saline (PBS) to a concentration within the detection range of the ELISA kit. The ELISA was performed using commercially available IL-6 and COX-2 ELISA kits. The microplate wells were coated with a primary antibody specific for IL-6 and COX-2, and the homogenates were added to the wells and incubated. After washing the wells, a secondary antibody conjugated with horseradish peroxidase (HRP) was added into the wells and incubated again. The substrate solution was added, and the reaction was stopped with a stop solution. The absorbance of the wells was measured at the appropriate wavelength using a microplate reader. The concentration of IL-6 and COX-2 in the homogenates was determined by comparing the absorbance values to the standard curve of recombinant IL-6 and COX-2 provided with the ELISA kit.

#### 2.9.22. In Vivo Anti-inflammatory Activity

The in vivo anti-inflammatory activity of the synthesized compounds **Ox-6a-f** was carried out by following the carrageenan-induced paw edema model in rats [48]. After assessing the safety profile of test compounds **Ox-6a-f** via acute toxicity in Sprague-Dawley rats (six weeks old) weighing about 150–200 g, random grouping into nine groups containing three rats each was done. Rats had free access to the laboratory feed and water. The National Institute of Health (NIH) guidelines were strictly carried out using test animals for experimentation. The Ethical Committee of Quaid-i-Azam University Islamabad approved the study protocol **(Bch#0267)** for animal care and experimentation. Group I: (Saline Control) normal saline was injected in the right hind paw and group rats were fed with fresh water and routine food. Group II: (Carrageenan Control) 1 mL/kg body weight of carrageenan solution (0.9% *w/v*; saline) was injected in the right hind paw and group rats were fed with fresh water and routine food. Group III: (Positive Control) rats orally received 10 mg/kg Ibuprofen (1:1 w/v DMSO) 1 h prior to 1 mL/kg body weight of carrageenan injection in the hind paw. Group IV: **OX-6a** rats orally received 10 mg/kg **OX-6a** (1:1 *w*/*v* DMSO) 1 h prior to 1 mL/kg body weight of carrageenan injection in the hind paw. Group V: **OX-6b** rats orally received 10 mg/kg **OX-6b** (1:1 *w*/*v* DMSO) 1 h prior to 1 mL/kg body weight of carrageenan injection in the hind paw. Group VI: **OX-6c** rats orally received 10 mg/kg **OX-6c** (1:1 *w*/*v* DMSO) 1 h prior to 1 mL/kg body weight of carrageenan injection in the hind paw. Group VII: **OX-6d** rats orally received 10 mg/kg **OX-6d** (1:1 *w*/*v* DMSO) 1 h prior to 1 mL/kg body weight of carrageenan injection in the hind paw. Group VIII: (**OX-6e**) rats orally received 10 mg/kg **OX-6e** (1:1 *w*/*v* DMSO) 1 h prior to 1 mL/kg body weight of carrageenan injection in the hind paw. Group IX: **OX-6f** rats orally received 10 mg/kg **OX-6f** (1:1 *w*/*v* DMSO) 1 h prior to 1 mL/kg body weight of carrageenan injection in the hind paw. Paw volume was measured using the Plethysmometer before dosing and at 1 h, 3 h, 6 h, and 12 h after carrageenan injection, and the following formulae for calculating the percent inhibition of edema was used:

EV = PVA − PVI

where, EV = Edema volume, PVI = initial paw volume (i.e., Paw volume before carrageenan administration), and PVA = Paw volume after carrageenan administration.

$$Percentage\;inhibition=\frac{\left(\mathrm{EVc}-\mathrm{EVt}\right)}{\mathrm{EVc}}\times 100$$

EVc = Edema volume of control animals, EVt = Edema volume of test sample animals.

## 3. Biochemical Analysis

### 3.1. Measurement of Endogenous Antioxidants

The anesthesia was induced in animals by injecting xylazine and ketamine (16 mg + 60 mg, i.p.) to prevent any discomfort. The animals were then euthanized at the end of the experiment by cervical dislocation, and paw tissues from different animal groups were homogenized. Then, potassium phosphate buffer (100 mM) and EDTA (1 mM) were added at pH 7.4. The mixture was then centrifuged at 12,000 rpm at 4 °C for 30 min. The concentrations of catalase (CAT), peroxidase (POD), superoxide dismutase (SOD), and glutathione (GSH) were measured from the supernatant by following the reported method [49]. The activities of CAT, POD, and SOD were determined by measuring the rate of H_2_O_2_ hydrolysis at 240 nm per minute. The change in absorbance of 0.01 units per minute is equal to catalase activity and is expressed as unit per milligram protein (U/mg protein). GSH was calculated at 405 nm as serum oxidation with DTNB (μM/mg protein) [50].

### 3.2. Measurement of Inflammatory Markers

The amount of nitric oxide (NO), lipid peroxidation (TBARS), interleukin-6 (IL-6), and cyclooxygenase-2 (COX-2) were determined in supernatants of homogenized rat paw tissue [51]. Briefly, the nitric oxide was determined as the reduction of the Griess reagent at a wavelength of 540 nm. The nitric oxide quantification (μM/mg protein) in serum was assessed by following the sodium nitrate curve. The TBARS was analyzed as nM TBARS/min/mg at a wavelength of 535 nm in the presence of the thiobarbituric acid reagent. The molar extinction coefficient (1.56 × 10^5^/M/cm) was used to measure lipid peroxidation at 37 °C. Pro-inflammatory cytokines *viz* IL-6 and COX-2 were determined in serum using ELISA kits by following the manufacturer’s instructions (ThermoFisher Scientific, Waltham, MA, USA) [52].

### 3.3. Statistical Analysis

The complete data in the present paper is reported as mean ± SD, and to ensure the variation in groups, a one-way analysis of variance was performed by using Statistix 8.1. Different graphs were plotted using Graph Pad Prim 8. Selecting confidence levels at 95% and 99%, i.e., *p* < 0.05 and *p* < 0.01, Tukey’s multiple comparison was performed for variations in data sets.

## 4. Conclusions

The 1,3,4-oxadiazole derivatives **Ox-6a-f** have been successfully synthesized in good yields. The in silico molecular docking results inferred that most of the synthesized compounds bind well with an active binding site of COX-2 compared to COX-1. The compound **Ox-6f** showed excellent binding affinity with a binding energy value (7.70 kcal/mol) among the synthesized derivatives. The Ramachandran plot confirms the presence of most residues of target proteins in the favored region, while MD simulation assured the stability of the protein–ligand complex of **Ox-6f** with the COX-2 protein. The antioxidant activities of the synthesized compounds have been performed to determine their free radical scavenging activity, including DPPH, OH, nitric oxide (NO), and iron chelation assay. The derivative **Ox-6f** showed promising results with 80.23% radical scavenging potential at a dose of 100 µg/mL, while ascorbic acid exhibited 87.72% inhibition at the same dose. The determination of inflammatory markers such as thiobarbituric acid-reducing substance, nitric oxide, interleikin-6 (IL-6), and COX-2 also assured the high anti-inflammatory potential of synthesized derivatives. The derivatives **Ox-6d** and **Ox-6f** displayed higher anti-inflammatory activity, exhibiting 70.56% and 74.16% activity, respectively. The results were compared with standard ibuprofen, which showed 84.31% activity at the same dose of 200 µg/mL. The in vivo anti-inflammatory activity of derivative **Ox-6f** was found to be excellent, showing 79.83% reduction in edema volume compared to standard ibuprofen, which exhibited 84.31% reduction of inflammation. As dry lab and wet lab results confirm each other, it has been deduced that derivative **Ox-6f** may serve as the lead structure to design potent compounds to address oxidative stress.

## Figures and Tables

**Table 1:** Docking results of oxadiazole derivatives **Ox-6a-f** against COX-2 and COX-1 using AutoDock.

Compounds	Binding Energy (kcal/mol) (COX-2) (PDBID 5KIR)	Binding Energy (kcal/mol) (COX-1) (PDBID 6Y3C)	Binding Energy (kcal/mol) (NOX) (PDBID 7U8G)
**Ox-6a**	−7.10	−6.30	−7.10
**Ox-6b**	−6.40	−6.10	−6.70
**Ox-6c**	−6.60	−6.20	−7.40
**Ox-6d**	−7.30	−6.70	−7.90
**Ox-6e**	−6.90	−6.40	−7.50
**Ox-6f**	−7.70	−6.80	−8.10

**Table 2:** Effects of oxadiazole derivatives **Ox-6a-f** on carrageenan-induced paw edema in rats.

Group	1st h	3rd h	6th h	12th h
**Saline Control**	--	--	--	--
**Carrageenan Control**	--	--	--	--
**Ibuprofen**	36.87 ± 4.21	54.03 ± 2.79	72.58 ± 1.82	84.71 ± 2.78 ^a^
**Ox-6a**	13.96 ± 8.61	36.82 ± 8.47	56.14 ± 4.72	74.52 ± 3.97 ^d^
**Ox-6b**	10.05 ± 2.56	21.51 ± 4.92	52.55 ± 1.82	57.96 ± 4.17 ^e^
**Ox-6c**	16.75 ± 8.22	23.38 ± 5.03	38.18 ± 4.94	51.59 ± 1.91 ^f^
**Ox-6d**	8.93 ± 3.48	30.11 ± 3.35	65.41 ± 2.04	76.64 ± 3.21 ^c^
**Ox-6e**	7.26 ± 3.48	26.07 ± 2.83	40.45 ± 2.04	52.44 ± 4.51 ^f^
**Ox-6f**	9.49 ± 1.67	29.31 ± 1.86	57.65 ± 1.73	79.83 ± 4.04 ^b^

Data values shown represent mean ± SD (n = 3). Means with different superscript (a–f) letters in the column are significantly (*p* < 0.05) different from one another.

**Table 3:** Effect of oxadiazole derivatives **Ox-6a-f** on antioxidant enzymes.

Groups	CAT (U/min)	POD (U/min)	SOD (U/min)	GSH (μM/mg Protein)
**Saline Control**	4.36 ± 1.28	8.03 ± 1.48	16.78 ± 1.21	23.33 ± 1.21
**Carrageenan Control**	2.93 ± 0.35	4.37 ± 0.94	09.39 ± 2.32	12.56 ± 1.53
**Ibuprofen**	4.41 ± 0.36	7.96 ± 1.04	15.06 ± 1.23	22.46 ± 0.21
**Ox-6a**	4.53 ± 0.55 ^a^	8.21 ± 1.33	15.94 ± 0.34 ^a^	21.86 ± 1.63 ^a^
**Ox-6b**	5.06 ± 0.95 ^a^	7.46 ± 0.32 ^a^	12.53 ± 2.29 ^a^	18.15 ± 0.58 ^a^
**Ox-6c**	5.53 ± 0.61 ^a^	7.13 ± 1.05 ^a^	13.03 ± 1.13 ^ab^	17.24 ± 2.31 ^a^
**Ox-6d**	5.31 ± 0.87 ^a^	8.23 ± 0.73 ^a^	16.43 ± 0.65 ^b^	21.52 ± 1.66 ^a^
**Ox-6e**	5.43 ± 0.81 ^b^	7.81 ± 1.51 ^b^	11.61 ± 0.92 ^c^	16.27 ± 0.79 ^b^
**Ox-6f**	4.66 ± 0.65	8.66 ± 0.41 ^ab^	15.74 ± 1.97 ^a^	21.72 ± 0.89 ^a^

**Note:** All the data are represented as mean ± SD (n = 3). Means with different superscript letters (^a^,^b^) in a column specify significant difference at (*p* < 0.05). CAT, catalase, POD, peroxidase, SOD, Superoxide dismutase, and GSH, reduced glutathione.

## Data Availability

The data presented in this study are available in insert article or supplementary material here.

## Supplementary Materials

The following supporting information can be downloaded at: https://www.mdpi.com/article/10.3390/ph16071045/s1. Figure S1. Ramachandran plot of COX-2 protein with PDBID 5KIR. Figure S2. Ramachandran plot of COX-1 protein with PDBID 6Y3C. Figure S3. Ramachandran plot of NOX protein with PDBID 7U8G.
