# Supplementary material for: Synthesis, Computational Studies, Antioxidant and Anti-Inflammatory Bio-Evaluation of 2,5-Disubstituted-1,3,4-Oxadiazole Derivatives
Source: Pharmaceuticals (Basel). 2023 Jul 24;16(7):1045. doi: 10.3390/ph16071045 (PMC10384447; doi:10.3390/ph16071045)
Supplement: Supplementary file 1 [file pharmaceuticals-16-01045-s001.zip › pharmaceuticals-2465876-supplementary.pdf]

Supporting Information

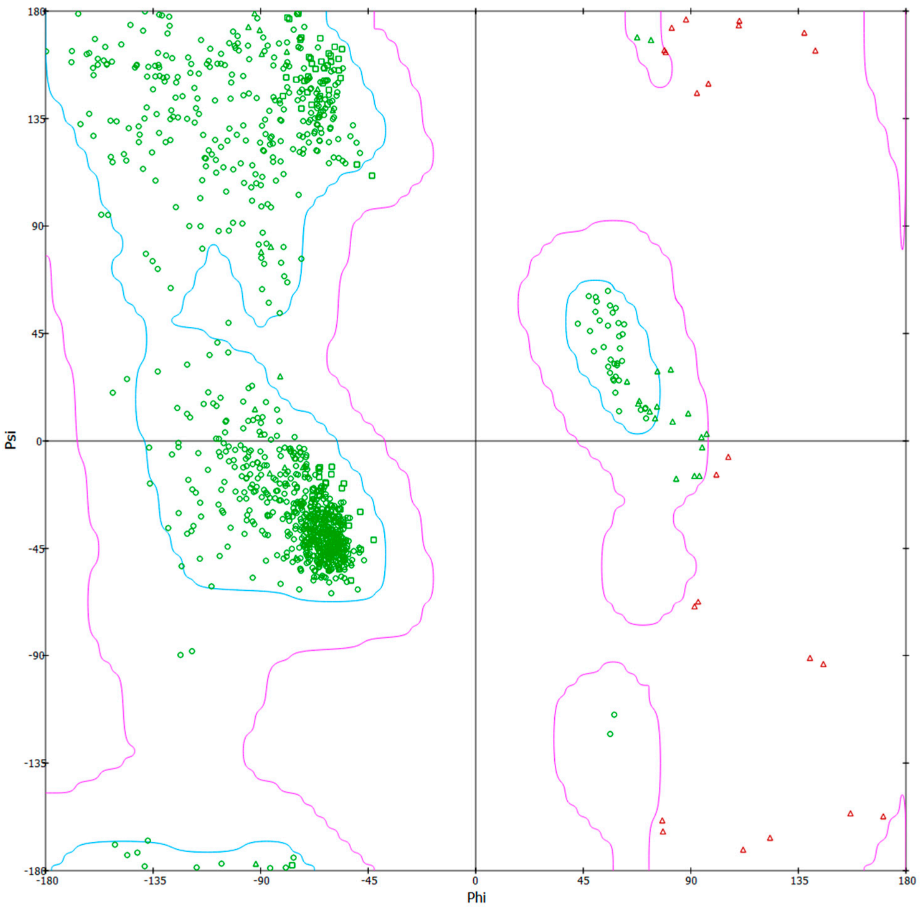

Figure S1. Ramachandran plot of COX-2 protein with PDBID 5KIR.

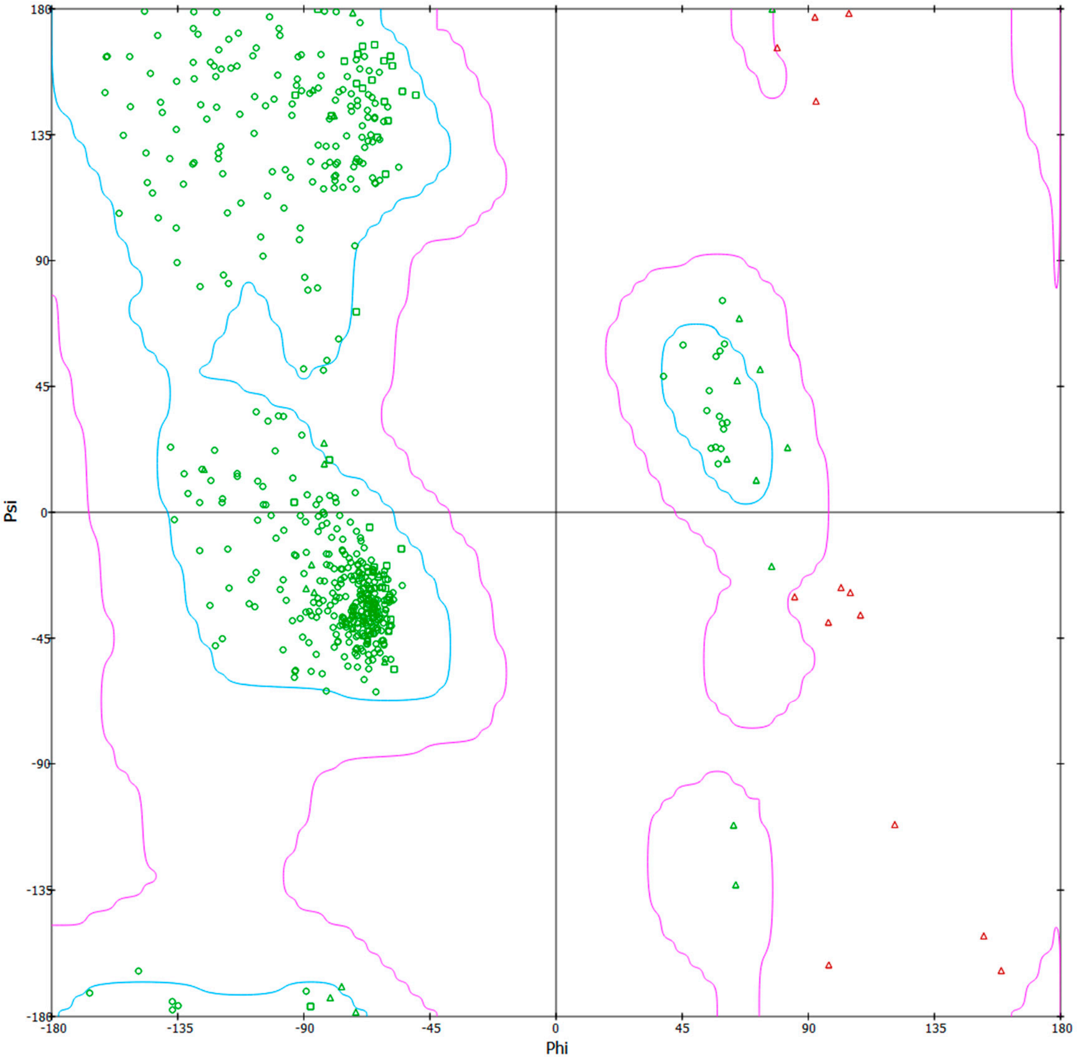

Figure S2. Ramachandran plot of COX-1 protein with PDBID 6Y3C.

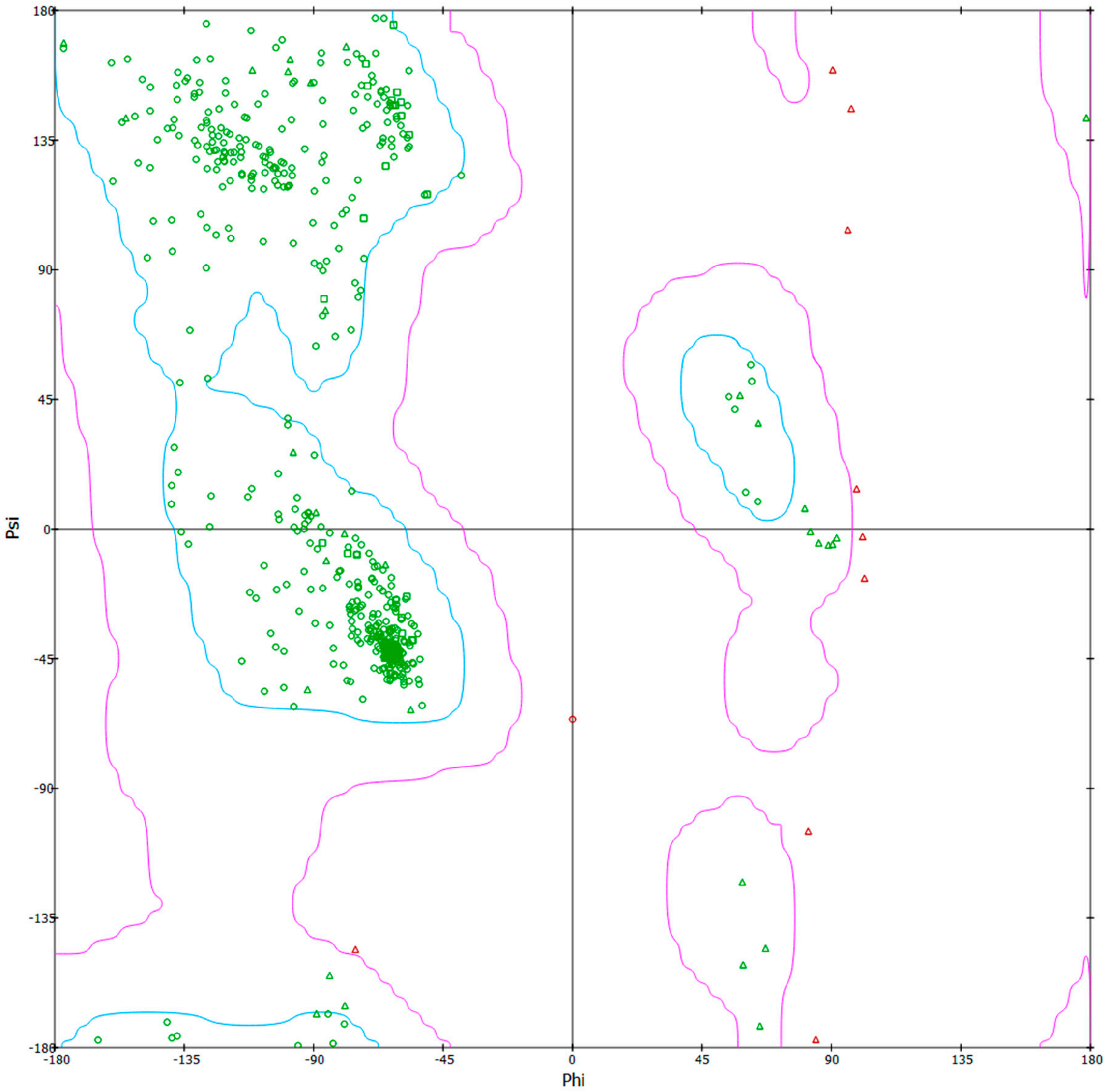

Figure S3. Ramachandran plot of NOX protein with PDBID 7U8G.

^{1}H and ^{13}C NMR Spectra of the synthesized compounds Ox-1-Ox-6

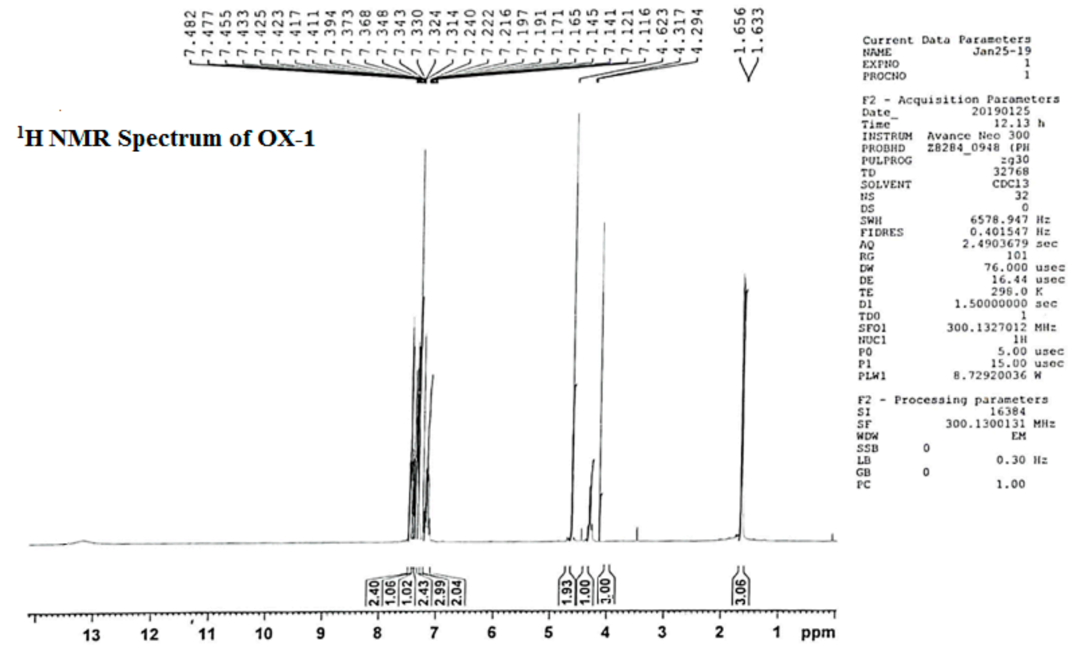

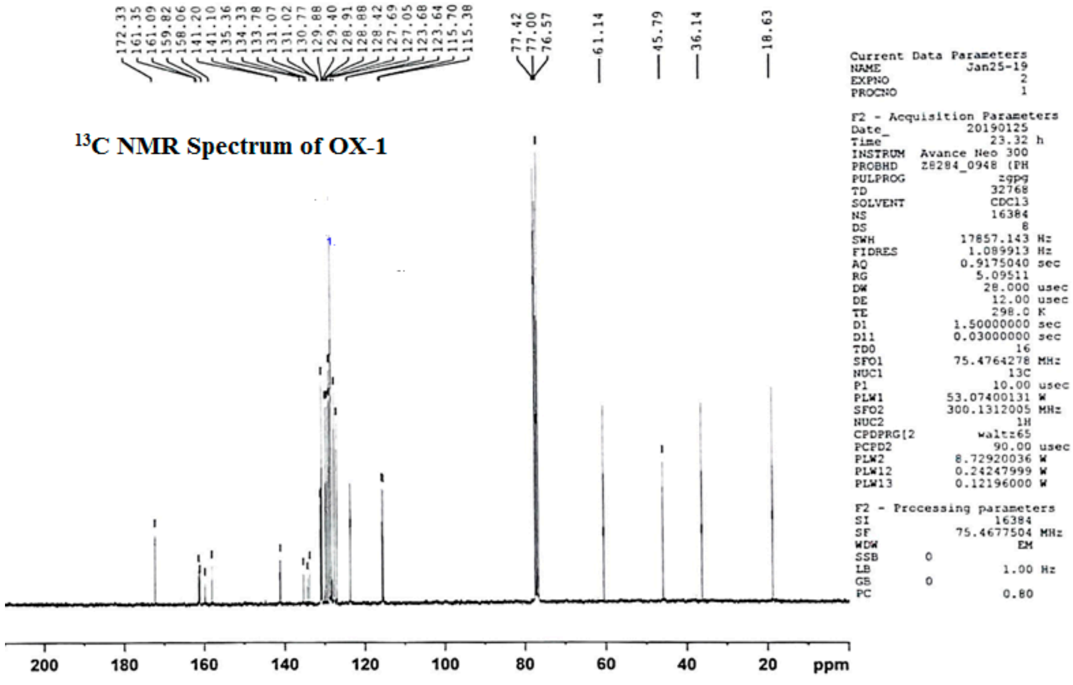

^{1}H NMR Spectrum of OX-2

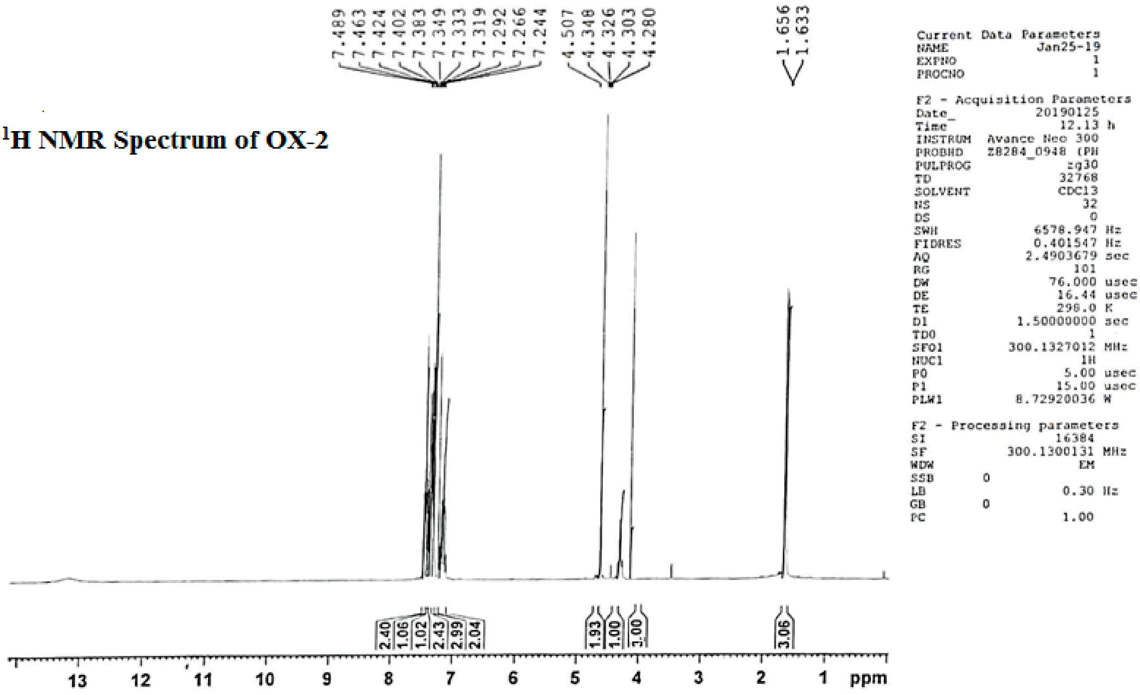

^{13}C NMR Spectrum of OX-2

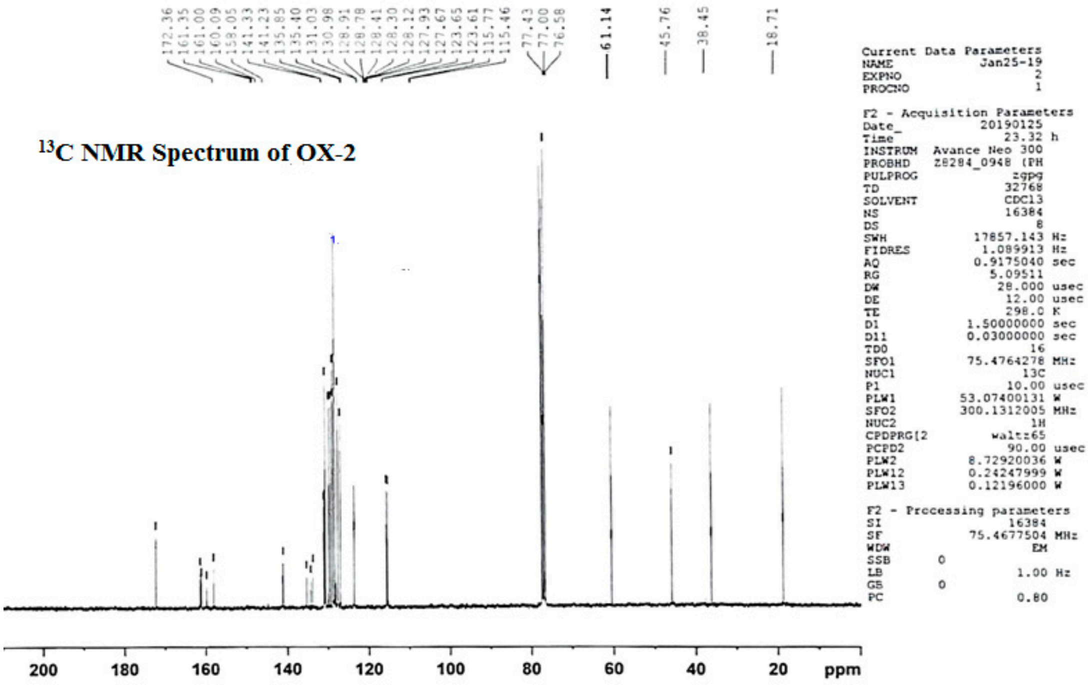

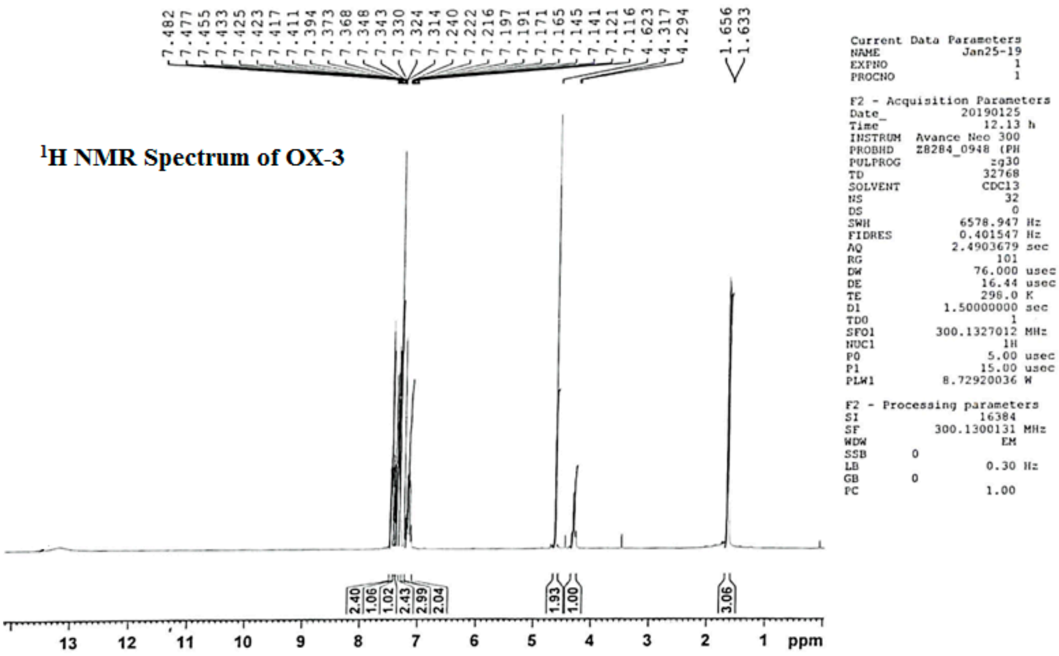

^{1}H NMR Spectrum of OX-3

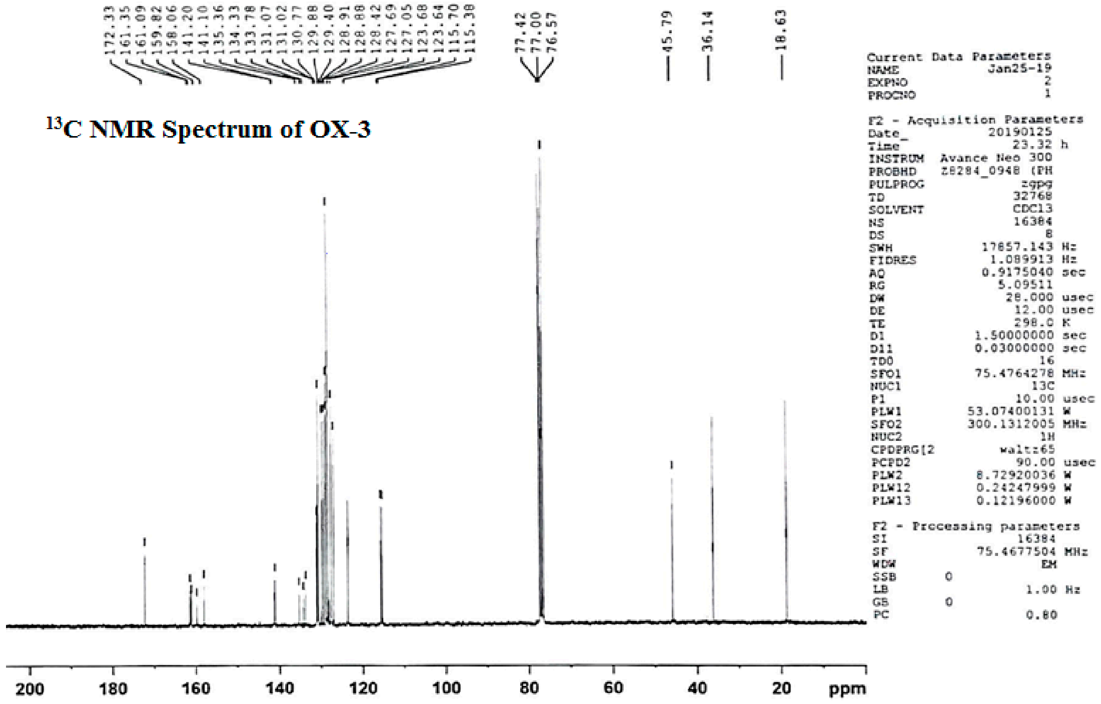

^{13}C NMR Spectrum of OX-3

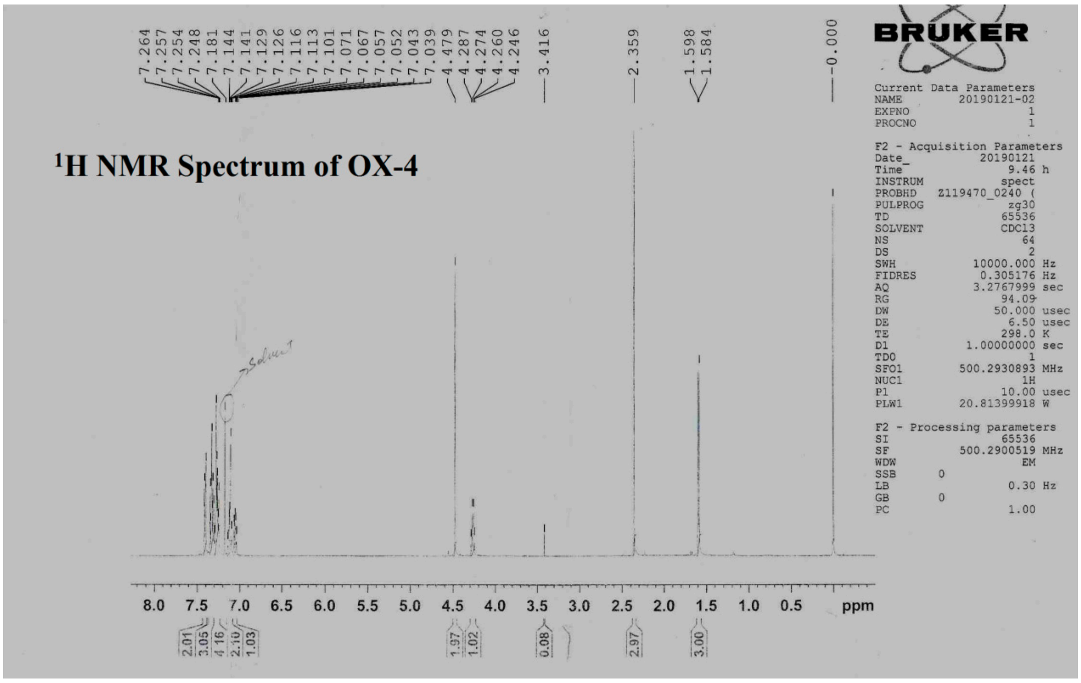

1H NMR Spectrum of OX-4
7.264 7.257 7.254 7.248 7.181 7.144 7.141 7.129 7.126 7.116 7.113 7.101 7.071 7.067 7.057 7.052 7.043 7.039 4.479 4.287 4.274 4.260 4.246 3.416 2.359 1.598 1.584 0.000
Solvent
8.0 7.5 7.0 6.5 6.0 5.5 5.0 4.5 4.0 3.5 3.0 2.5 2.0 1.5 1.0 0.5 ppm
2.01 3.05 4.16 2.10 1.03 1.97 1.02 0.08 2.97 3.00
BRUKER
Current Data Parameters
NAME 20190121-02
EXPNO 1
PROCNO 1
F2 - Acquisition Parameters
Date_ 20190121
Time 9.46 h
INSTRUM spect
PROBHD Z119470_0240 (
PULPROG zg30
TD 65536
SOLVENT CDCl3
NS 64
DS 2
SWH 10000.000 Hz
FIDRES 0.305176 Hz
AQ 3.2767999 sec
RG 94.09
DW 50.000 usec
DE 6.50 usec
TE 298.0 K
D1 1.00000000 sec
TD0 1
SFO1 500.2930893 MHz
NUC1 1H
P1 10.00 usec
PLW1 20.81399918 W
F2 - Processing parameters
SI 65536
SF 500.2900519 MHz
WDW EM
SSB 0
LB 0.30 Hz
GB 0
PC 1.00

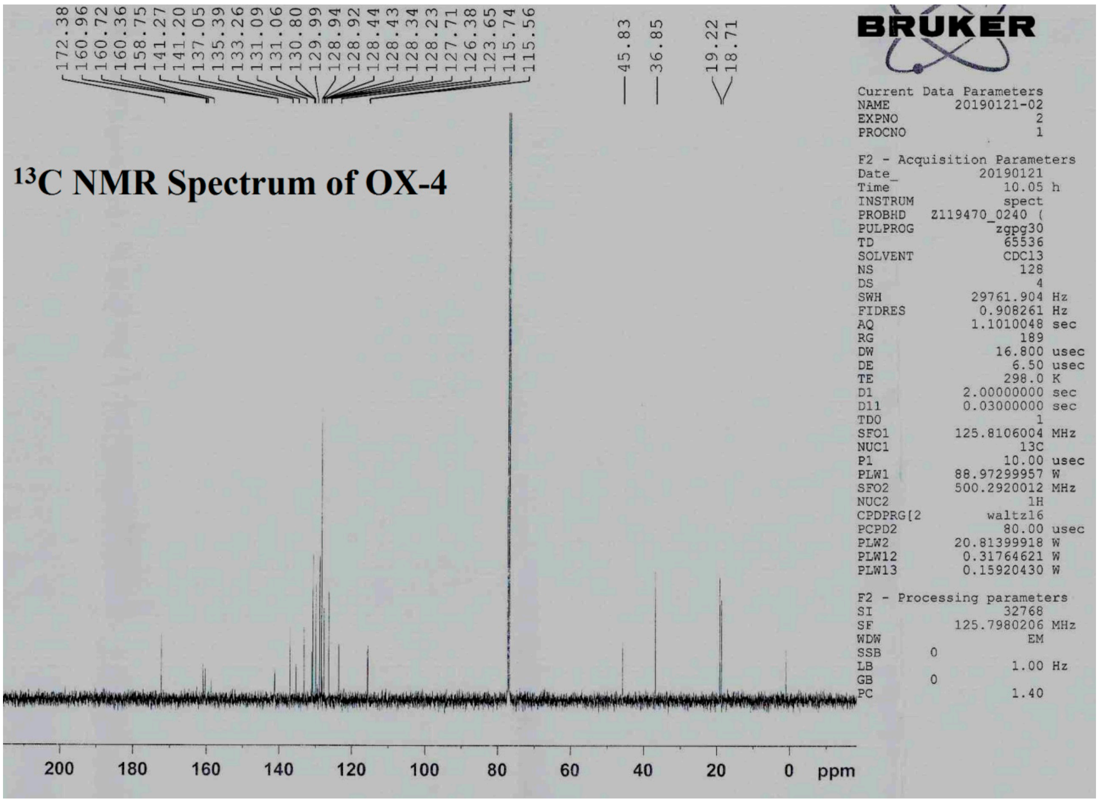

13C NMR Spectrum of OX-4
172.38 160.96 160.72 160.36 158.75 141.27 141.20 137.05 135.39 133.26 131.09 131.06 130.80 129.99 128.94 128.92 128.44 128.43 128.34 128.23 127.71 126.38 123.65 115.74 115.56 45.83 36.85 19.22 18.71
200 180 160 140 120 100 80 60 40 20 0 ppm
BRUKER
Current Data Parameters
NAME 20190121-02
EXPNO 2
PROCNO 1
F2 - Acquisition Parameters
Date_ 20190121
Time 10.05 h
INSTRUM spect
PROBHD Z119470_0240 (
PULPROG zgpg30
TD 65536
SOLVENT CDCl3
NS 128
DS 4
SWH 29761.904 Hz
FIDRES 0.908261 Hz
AQ 1.1010048 sec
RG 189
DW 16.800 usec
DE 6.50 usec
TE 298.0 K
D1 2.00000000 sec
D11 0.03000000 sec
TD0 1
SFO1 125.8106004 MHz
NUC1 13C
P1 10.00 usec
PLW1 88.97299957 W
SFO2 500.2920012 MHz
NUC2 1H
CPDPRG[2 waltz16
PCPD2 80.00 usec
PLW2 20.81399918 W
PLW12 0.31764621 W
PLW13 0.15920430 W
F2 - Processing parameters
SI 32768
SF 125.7980206 MHz
WDW EM
SSB 0
LB 1.00 Hz
GB 0
PC 1.40

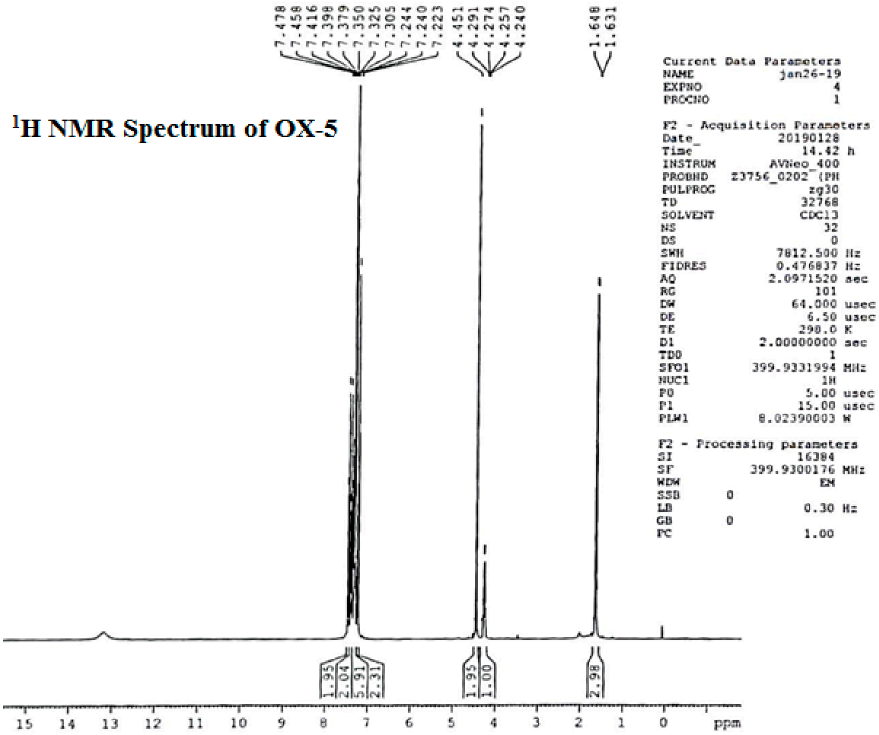

1H NMR Spectrum of OX-5
7.478 7.458 7.416 7.398 7.379 7.350 7.325 7.305 7.244 7.240 7.223
4.451 4.291 4.274 4.257 4.240
1.648 1.631
Current Data Parameters
NAME jan26-19
EXPNO 4
PROCNO 1
F2 - Acquisition Parameters
Date_ 20190128
Time 14.42 h
INSTRUM AVNeo_400
PROBHD Z3756_0202_(PH
PULPROG zg30
TD 32768
SOLVENT CDCl3
NS 32
DS 0
SWH 7812.500 Hz
FIDRES 0.476837 Hz
AQ 2.0971520 sec
RG 101
DW 64.000 usec
DE 6.50 usec
TE 298.0 K
D1 2.00000000 sec
TD0 1
SFO1 399.9331994 MHz
NUC1 1H
P0 5.00 usec
P1 15.00 usec
PLW1 8.02390003 W
F2 - Processing parameters
SI 16384
SF 399.9300176 MHz
WDW EM
SSB 0
LB 0.30 Hz
GB 0
PC 1.00
1.95 2.04 5.91 2.31 1.95 1.00 2.98
15 14 13 12 11 10 9 8 7 6 5 4 3 2 1 0 ppm

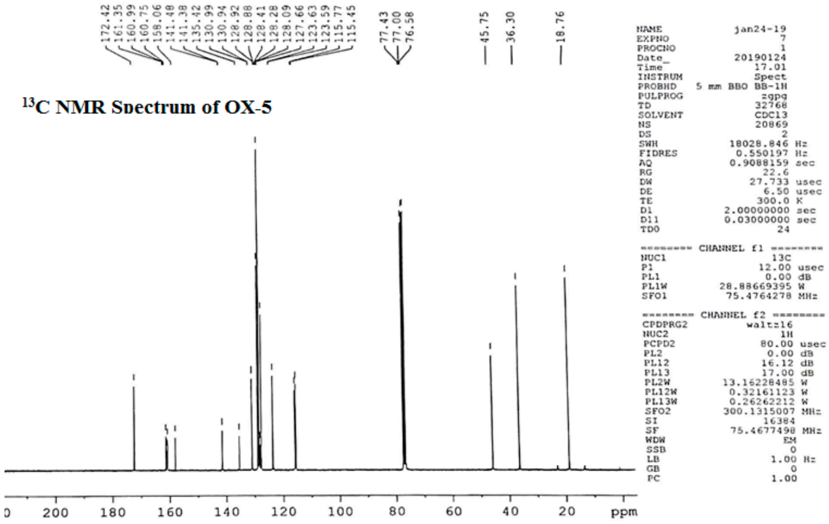

13C NMR Spectrum of OX-5
172.42 161.35 160.99 160.75 158.06 141.48 141.38 135.42 130.99 130.94 128.92 128.88 128.41 128.28 128.09 127.66 123.63 123.59 115.77 115.45
77.43 77.00 76.58
45.75
36.30
18.76
NAME jan24-19
EXPNO 7
PROCNO 1
Date_ 20190124
Time 17.01
INSTRUM Spect
PROBHD 5 mm BBO BB-1H
PULPROG zgpg
TD 32768
SOLVENT CDCl3
NS 20869
DS 2
SWH 18028.846 Hz
FIDRES 0.550197 Hz
AQ 0.9088159 sec
RG 22.6
DW 27.733 usec
DE 6.50 usec
TE 300.0 K
D1 2.00000000 sec
D11 0.03000000 sec
TD0 24
======== CHANNEL f1 ========
NUC1 13C
P1 12.00 usec
PL1 0.00 dB
PL1W 28.88669395 W
SFO1 75.4764278 MHz
======== CHANNEL f2 ========
CPDPRG2 waltz16
NUC2 1H
PCPD2 80.00 usec
PL2 0.00 dB
PL12 16.12 dB
PL13 17.00 dB
PL2W 13.16228485 W
PL12W 0.32161123 W
PL13W 0.26262212 W
SFO2 300.1315007 MHz
SI 16384
SF 75.4677498 MHz
WDW EM
SSB 0
LB 1.00 Hz
GB 0
PC 1.00
200 180 160 140 120 100 80 60 40 20 ppm

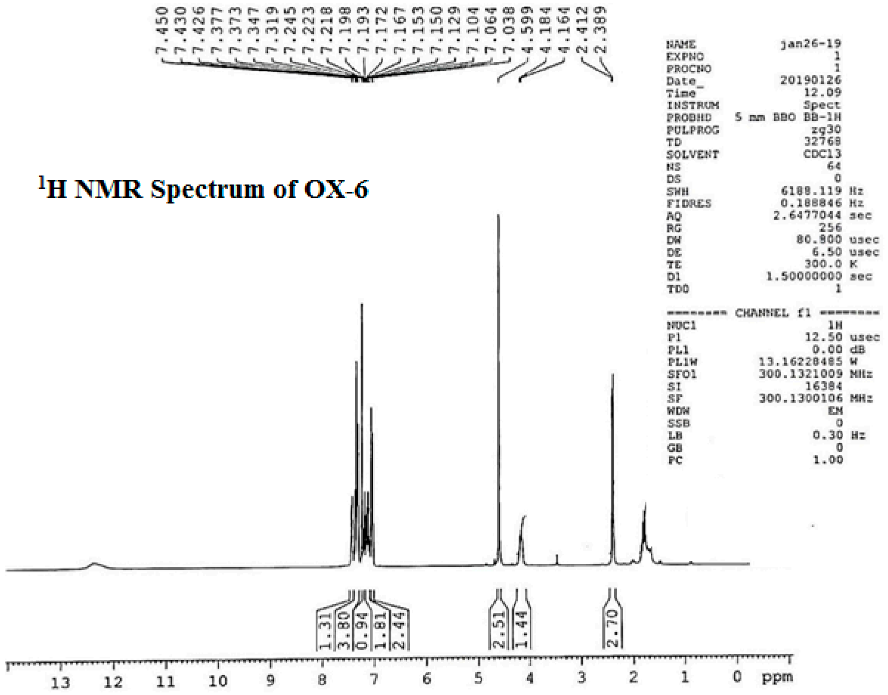

7.450
7.430
7.426
7.377
7.373
7.347
7.319
7.245
7.223
7.218
7.198
7.193
7.172
7.167
7.153
7.150
7.129
7.104
7.064
7.038
4.599
4.184
4.164
2.412
2.389
NAME jan26-19
EXPNO 1
PROCNO 1
Date_ 20190126
Time 12.09
INSTRUM Spect
PROBHD 5 mm BBO BB-1H
PULPROG zg30
TD 32768
SOLVENT CDCl3
NS 64
DS 0
SWH 6188.119 Hz
FIDRES 0.188846 Hz
AQ 2.6477044 sec
RG 256
DW 80.800 usec
DE 6.50 usec
TE 300.0 K
D1 1.50000000 sec
TD0 1
======== CHANNEL f1 ========
NUC1 1H
P1 12.50 usec
PL1 0.00 dB
PL1W 13.16228485 W
SFO1 300.1321009 MHz
SI 16384
SF 300.1300106 MHz
WDW EM
SSB 0
LB 0.30 Hz
GB 0
PC 1.00
1H NMR Spectrum of OX-6
1.31
3.80
0.94
1.81
2.44
2.51
1.44
2.70
13 12 11 10 9 8 7 6 5 4 3 2 1 0 ppm

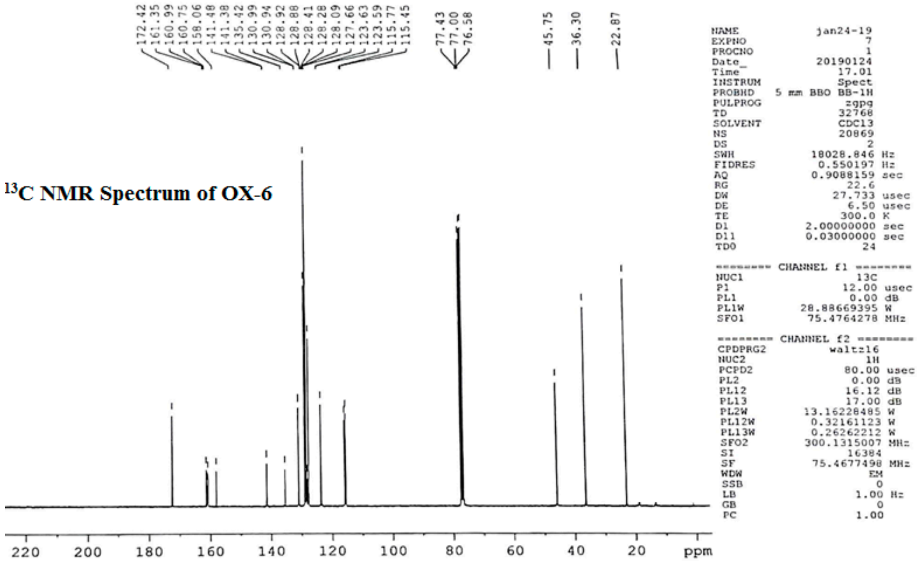

172.42
161.35
160.99
160.75
158.06
141.48
141.38
135.42
130.99
130.94
128.92
128.88
128.41
128.28
128.09
127.66
123.63
123.59
115.77
115.45
77.43
77.00
76.58
45.75
36.30
22.87
NAME jan24-19
EXPNO 7
PROCNO 1
Date_ 20190124
Time 17.01
INSTRUM Spect
PROBHD 5 mm BBO BB-1H
PULPROG zgpg
TD 32768
SOLVENT CDCl3
NS 20869
DS 2
SWH 18028.846 Hz
FIDRES 0.550197 Hz
AQ 0.9088159 sec
RG 22.6
DW 27.733 usec
DE 6.50 usec
TE 300.0 K
D1 2.00000000 sec
D11 0.03000000 sec
TD0 24
======== CHANNEL f1 ========
NUC1 13C
P1 12.00 usec
PL1 0.00 dB
PL1W 28.88669395 W
SFO1 75.4764278 MHz
======== CHANNEL f2 ========
CPDPRG2 waltz16
NUC2 1H
PCPD2 80.00 usec
PL2 0.00 dB
PL12 16.12 dB
PL13 17.00 dB
PL2W 13.16228485 W
PL12W 0.32161123 W
PL13W 0.26262212 W
SFO2 300.1315007 MHz
SI 16384
SF 75.4677498 MHz
WDW EM
SSB 0
LB 1.00 Hz
GB 0
PC 1.00
13C NMR Spectrum of OX-6
220 200 180 160 140 120 100 80 60 40 20 ppm
